# In Situ and Operando Characterization Techniques in Stability Study of Perovskite-Based Devices

**DOI:** 10.3390/nano13131983

**Published:** 2023-06-30

**Authors:** Bingchen He, Chenyue Wang, Jielei Li, Zhenhuang Su, Guichuan Xing, Xingyu Gao, Shi Chen

**Affiliations:** 1 Joint Key Laboratory of the Ministry of Education, Institute of Applied Physics and Materials Engineering, University of Macau, Avenida da Universidade, Taipa, Macau 999078, China; 2 Shanghai Synchrotron Radiation Facility (SSRF), Shanghai Advanced Research Institute, Chinese Academy of Sciences, Shanghai 201204, China

**Keywords:** in situ/operando, perovskite materials, degradation

## Abstract

Metal halide perovskite materials have demonstrated significant potential in various optoelectronic applications, such as photovoltaics, light emitting diodes, photodetectors, and lasers. However, the stability issues of perovskite materials continue to impede their widespread use. Many studies have attempted to understand the complex degradation mechanism and dynamics of these materials. Among them, in situ and/or operando approaches have provided remarkable insights into the degradation process by enabling precise control of degradation parameters and real-time monitoring. In this review, we focus on these studies utilizing in situ and operando approaches and demonstrate how these techniques have contributed to reveal degradation details, including structural, compositional, morphological, and other changes. We explore why these two approaches are necessary in the study of perovskite degradation and how they can be achieved by upgrading the corresponding ex situ techniques. With recent stability improvements of halide perovskite using various methods (compositional engineering, surface engineering, and structural engineering), the degradation of halide perovskite materials is greatly retarded. However, these improvements may turn into new challenges during the investigation into the retarded degradation process. Therefore, we also highlight the importance of enhancing the sensitivity and probing range of current in situ and operando approaches to address this issue. Finally, we identify the challenges and future directions of in situ and operando approaches in the stability research of halide perovskites. We believe that the advancement of in situ and operando techniques will be crucial in supporting the journey toward enhanced perovskite stability.

## 1. Introduction

In the past decade, there has been significant research focus on halide perovskite materials for various optoelectronic devices, including photovoltaics [1], light emitting diode [2,3,4], photodetectors [5], lasers [6], and so on. The general chemical formula of halide perovskite materials is ABX_3_, where the A site can be occupied by organic cations, such as methyl ammonium (MA), formamidinium (FA), or inorganic cation cesium. The B site can be divalent cations, such as lead(Pb) or tin(Sn), while the X site consists of halide anions, such as chlorine(Cl), bromine(Br) or iodine(I). Due to the great variety of its chemical composition, halide perovskite forms a big material family with tunable optoelectronic properties. The excellent optoelectronic properties of halide perovskite include its exceptional high absorption coefficient over a wide spectrum [7], a direct bandgap with excellent tunability in the visible–infrared range [8], long and balanced charge carrier diffusion lengths in the micrometer range [9,10], and defect tolerance [11,12]. The potential of perovskite materials is exemplified by the development of perovskite solar cells. An unprecedented rise in their efficiency from 3.8% to 25.7% was achieved in only a few years [13,14,15], which was comparable to traditional Si-based solar cells and outperformed all other emerging solar cells, e.g., organic, and CIGS-, CdTe-, and quantum-dot-based solar cells [16]. 

Currently, the major hurdle in halide perovskite research and applications is its intrinsic stability problem [17]. It is well known that halide perovskite is vulnerable to various environmental stresses, including water, heat, light, oxygen, and electric fields [18,19,20,21]. In early studies, this vulnerability was easily identified by a quick color change of the film and short device lifetime [22]. Later on, multiple characterization techniques, such as X-ray diffraction (XRD), scanning electron microscopy (SEM), X-ray photoelectron spectroscopy (XPS), atomic force microscopy (AFM), and so on, were used to study the degradation process in detail [23,24,25,26]. At first glance, the degradation of perovskite seems simple, but systematic studies found that the degradation is a complex process, which is highly dependent on the type of perovskite and the details of the exposure conditions. Therefore, it is important to control the exposure conditions and to monitor the degradation in real time. Keeping this complexity in mind, we could understand why studies with in situ and operando approaches reveals more degradation details than those studies performed in an ex situ manner. With the help of in situ and operando approaches, the degradation mechanism of 3D perovskite (MAPbI_3_ and FAPbI_3_) has been thoroughly studied [19,23,24]. It has been found that the degradation of 3D perovskite can be divided into three different categories: morphological change, ion migration, and decomposition. Morphological changes in perovskite materials are primarily due to recrystallization, which usually involves water. A morphological change can happen much earlier than decomposition [27]. However, it may damage the device integrity and be responsible for premature device failure. Ion migration is mainly driven by internal or external electrical fields during the operation of devices [28]. Ion migration threatens the long-term stability and cannot be alleviated by protection methods like encapsulation. The decomposition of perovskite is due to irreversible loss of certain components (mainly organic parts), causing permanent changes in perovskite materials. In general, all three degradation pathways can be accelerated when more than one stresses are present. In a real device, degradation is usually caused by the combination of all three categories, depending on the types and magnitude of degradation stresses it is facing.

In situ and operando are two approaches used in characterization techniques to study the perovskite degradation process. The concept of “in situ” is widely used in surface-related research aiming to study surface changes in an ultrahigh vacuum (UHV) condition to avoid ambient exposure. A precisely controlled environment helps to reveal the surface change that are specific to certain factors. In the study of perovskite degradation, an in situ approach is capable of revealing the degradation clearly due to a specific stress (such as water or heat). However, due to the constraints of a UHV condition, the “strength” of the stress is significantly limited (for water, an in situ study can only reach ~10^−5^ mbar). On the other hand, the concept of “operando” was first introduced by chemists in 2002 to study catalytical reactions in real time and in realistic conditions [29,30,31]. It a much wider reaction conditions than an in situ approach. In perovskite studies, an operando approach helps to investigate degradation under realistic conditions or beyond. For example, in water-induced degradation studies, an operando approach can reach 90% relative humidity (RH) at room temperature, which is equivalent to ~27 mbar, too high for any ultrahigh vacuum equipment [32]. The two approaches are complementary to each other because an in situ approach pinpoints the detailed mechanism under one of multiple degradation stresses while an operando approach reveals the real degradation dynamics in an environment much closer to a real environment.

Although there are many reviews focusing on the perovskite degradation [20,27,28], the importance of in situ and operando approaches in perovskite degradation studies has not been systematically reviewed yet. We would like to give three reasons to highlight the importance of these two approaches in details. First, many degradation studies of halide perovskite are based on in situ and/or operando approaches. Most of the reviews are focusing on degradation alone, with little attention on the techniques themselves. We use the first part of this review to summarize the technological modifications adopted in in situ and operando characterizations and explain how they help to reveal degradation mechanism. Second, most of current in situ and operando studies are not specialized for perovskite studies and therefore have great potential in further optimization. The unique requirements of halide perovskite on in situ and operando studies (e.g., high water humidity, light sensitive, pressure sensitive, electrical field sensitive, etc.) need to be considered so that a better experimental setup can be achieved. Last, to cope with future degradation studies, current in situ and operando approaches need to be further improved with a higher probing sensitivity and a wider range of degradation conditions. There are various protection methods to improve the stability of perovskite materials, but they is still far less stable than their inorganic counterparts (i.e., silicon) [33]. The effectiveness of these protection methods needs to be carefully examined from a fundamental point of view to further slow the degradation of these stabilized perovskite materials. Therefore, future in situ and operando techniques must overcome the following challenges: (1) slower degradation process due to protection/passivation; (2) harsher degradation conditions to accelerate degradation processes or aging tests, such as higher temperature, higher moisture, and higher electrical field, etc.; and (3) complex degradation conditions to include multiple degradation stresses, such as water/heat, water/light, light/electrical field and so on to simulate real challenges during device operation. In short, this review aims to summarize the achievements, challenges, and future advancements of in situ/operando approaches in perovskite stability studies.

**Figure 1:**
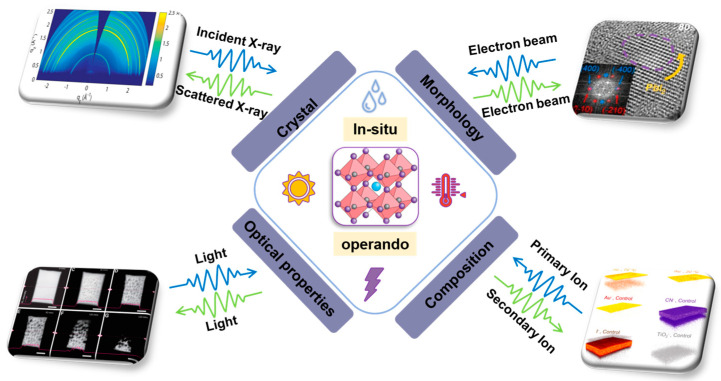
Schematics of the in situ and operando techniques to study halide perovskite stability [34,35,36]. Copyright 2016, American Chemical Society. Copyright 2017, Elsevier. Copyright 2018, Wiley.

We organized this review based on different characterization techniques. In each section, we will briefly mention the techniques themselves, followed by result comparison between normal and in situ/operando techniques. We conclude each section with potential advancements in the specific in situ/operando approaches. We start with structural detection techniques in Section 2, followed by composition techniques in Section 3. Morphological techniques and optoelectronic techniques will be in Section 4 and Section 5. In Section 6, we give an outlook on how to modify current in situ/operando techniques for future stability studies. We believe this review will serve as a technology milestone for in situ and operando studies in perovskite studies and provide important insights in these two approaches. The overall framework of this review is shown in Figure 1.

## 2. Crystal Structural Characterization Techniques

Crystallinity is a fundamental property of materials. In fact, the name of perovskite materials is from their crystal structure. Perovskite materials have black and yellow phases, where the black phase includes α, β, and γ structures, corresponding to cubic, tetragonal, and orthorhombic structures, respectively. The yellow phase refers to the δ structure (orthorhombic structure) [37,38,39]. It is well known that the crystal structure of perovskite depends on its composition and temperature [40,41]. For example, MAPbI_3_ has three crystal structures: cubic (>330 K), tetragonal (160 K~330 K), and orthorhombic (<160 K). Since the degradation of perovskite materials is commonly associated with structural change, many structural characterization techniques have been used in degradation study, such as X-ray diffraction (XRD), grazing incidence wide-angle X-ray scattering (GIWAXS), and grazing incidence neutron scattering (GISANS). These techniques are based on diffraction laws and are sensitive to the structural changes that occur during degradation. 

### 2.1. Perovskite Degradation Studies by In Situ/Operando X-ray Scattering Techniques

X-ray diffraction is the most widely used technique in crystal structure determination. It uses Bragg’s diffraction law to probe the long-range crystalline order in samples. In iodide perovskite studies, ex situ XRD typically uses the signal of PbI_2_ at 12.7° to infer the status of perovskite degradation or the quality of pristine perovskite [42,43]. In FAPbI_3_, XRD also provides its phase information because the photoactive α phase has a different diffraction peak (13.9°) from the photo inactive δ phase (11.9°) [44]. In addition, ex situ XRD can also confirm the existence of a metastable dihydrate (CH_3_NH_3_)_4_PbI_6_·2H_2_O if the perovskite were kept in the dark [42,43]. By using the ex situ XRD technique, perovskite degradation under different stresses, such as water, light, and heat, can be easily confirmed [20,22]. 

**Figure 2:**
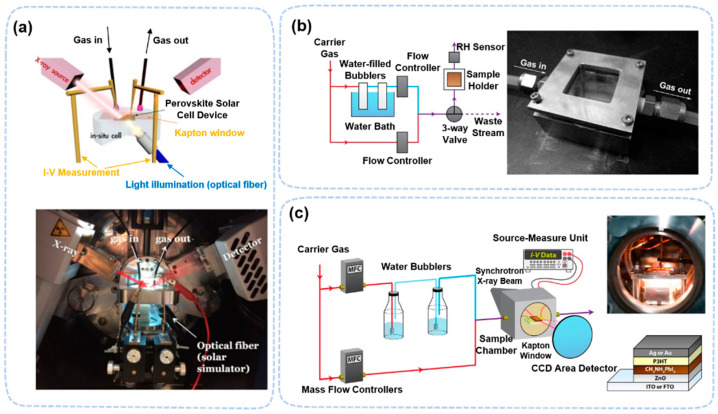
(**a**) Schematics and images of the cell configuration for in situ X-ray microscopy measurement [47]. Copyright 2017, American Chemical Society. (**b**) Schematic of the RH control apparatus and sample holder [45]. Copyright 2015, American Chemical Society. (**c**) Diagram of the humidity-control apparatus and in situ sample chamber [46]. Copyright 2018, American Chemical Society.

Because the hard X-ray used in XRD has a high penetration depth, there is no limit on pressure or other factors in the XRD measurement. Therefore, for this technique, in situ and operando approaches can be easily realized simultaneously. To perform in situ/operando XRD, the sample is usually kept in a commercially available flat airtight chamber with an X-ray window. The window material can be Kapton films. Other accessories, such as for gas regulation and temperature control, may be added to this chamber during in situ studies. For example, in water-induced degradation, a pump and gas inlet are connected to adjust the humidity level [45,46]. A humidity sensor is connected either inside the chamber or along the gas line to monitor the actual humidity level (Figure 2a–c). 

**Figure 3:**
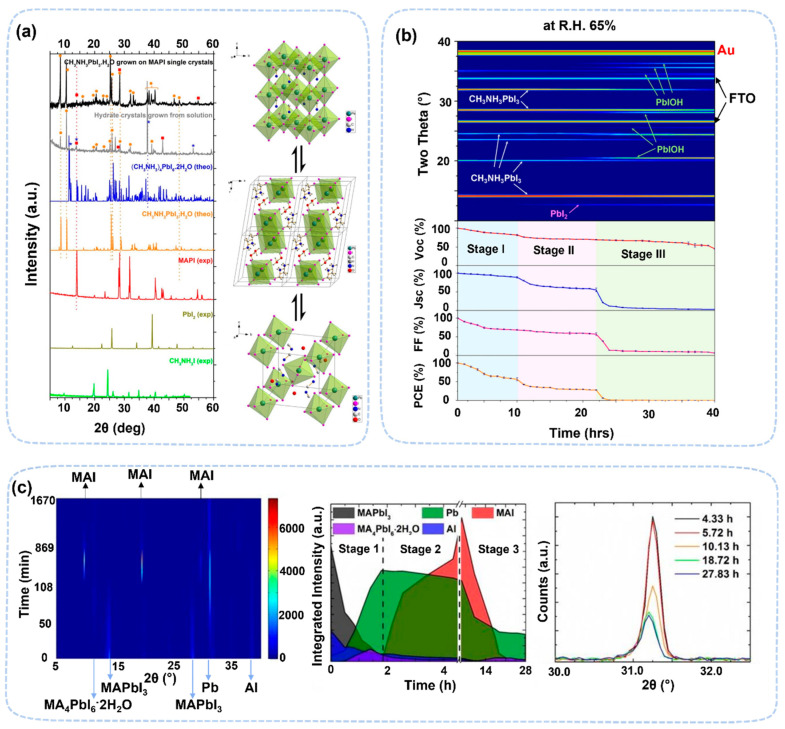
(**a**) In situ XRD patterns of MAPbI_3_: hydration of a MAPbI_3_ film and dihydration of a MAPbI_3_ film. Structural changes of MAPbI_3_ under high humidity conditions, from MAPbI_3_ to monohydrate phase and then to dihydrate [48]. Copyright 2015, American Chemical Society. (**b**) In situ X-ray diffraction measurement and the corresponding cell performance along time evolution at RH 65% [47]. Copyright 2017, American Chemical Society. (**c**) In situ XRD analysis of the degradation of the perovskite materials with moist air in the dark [32]. Copyright 2016, American Chemical Society.

The most notable discovery made from operando XRD is the revealing of the monohydrate and dihydrate phases in the water-induced degradation of MAPbI_3_. Their characteristic peaks are at 8.7° and 11.6°, respectively [48,49,50]. There are two influencing conditions for the formation of hydrate: humidity level and exposure time. Hydrates need higher-than-threshold humidity conditions (~70% RH) to form. It was also observed that the formation of hydrates can be shortened when the relative humidity is even higher. The monohydrate forms first, ~30 min at 80% RH, and the dihydrate phase forms later ~120 min at 80% RH condition. The monohydrate has a molecular formula of MAPbI_3_·H_2_O, and it can turn back to MAPbI_3_ under dry conditions (Figure 3a). The formation of monohydrate changes the perovskite structure from 3D to 1D. It incorporates one-dimensional, isolated [PbI_3_]^−^ double chains, forming a two-octahedra-wide “ribbon” that is similar to δ structures. As the exposure time to water conditions increases, the monohydrate turns into dihydrate, (CH_3_NH_3_)_4_PbI_6_·2H_2_O, in which each primary cell of perovskite includes two water molecules. This process is partially reversible due to PbI_2_ formation. The perovskite structure changes from 1D to 0D. The intermediate phases were found in devices when exposed to water [47]. The formation condition varies depending on the details of the device structure, which may be due to interfacial influences or local water aggregation, resulting in hydrate formation at low humidity. Chen et al. investigated the degradation process of FTO/TiO_2_/MAPbI_3_/spiro-OMeTAD/Au devices under ~65% RH humidity using operando XRD (Figure 3b) [47]. This degradation process can be divided into three stages. In the first stage, the perovskite material first forms hydrate CH_3_NH_3_PbI_3_·H_2_O, which leads to the loss of its fill factor. In the second stage, the hydrate is transformed into PbI_2_, which reduces the J_SC_ and V_OC_ values of the device. In the third stage, PbI_2_ decomposes to form PbIOH (~20.5°) due to the combined effect of light and moisture. operando XRD also observed structural changes due to water-induced ion migration. At 30% RH, Pb_0_ (31.2°) is formed due to a redox reaction in perovskite with Al electrodes (Figure 3c). In devices, perovskite films could form hydrates at RH as low as 30%, which is different for the degradation perovskite thin films (~70% RH) [48]. These results reveal that water molecules in devices could induce a redox reaction between electrode and perovskite, reducing the activation energy of ion migration and facilitating the diffusion of ions.

In heat-induced degradation, operando XRD could observe the composition change more closely. It was observed that MA-perovskite is less heat-stable than FA-perovskite. MA-perovskite decomposed at ~130 °C, while FA-perovskite decomposed at 160 °C. The mixed perovskite material (FA_0.83_Cs_0.17_PbI_2_Br) shows better thermal stability than the MA- and FA-perovskite. Its thermal decomposition process can be divided into three phases [51]. Below 160 °C, the crystal structure is *Pm3m*, and no phase change or significant decomposition occurs. Though the peak at 14.24° is slightly lower angle, it is thought that the change is caused by the expansion of the structure due to the loss of Br elements. When the temperature is higher than 160 °C, the perovskite decomposes into PbI_2_ due to the volatilization of FA ions and halogen elements. When the temperature rises to 220 °C, new substances, such as CsPbI_3−z_Br_z_ (z << 1) with *Pnma* structures, are formed. Temperature-induced decomposition is highly sensitive to the types of ions in perovskite. Tan et al. compared the thermal degradation processes of FA_0.83_Cs_0.17_Pb(I_0.83_Br_0.17_)_3_ and Cs_0.05_(MA_0.17_FA_0.83_)_0.95_Pb(I_0.83_Br_0.17_)_3_, by in situ XRD [52]. The experimental results show that the triple-cation perovskite material is less thermally stable than the dual-cation perovskite material because of the MA component. Prepared perovskite films containing MACl show better thermal stability than their counterpart without Cl^−^. In comparison to MAPbI_3_, perovskite materials with containing Cl^−^ can maintain the ABX_3_ structure at higher temperature (~300 °C) because the addition of Cl^−^ can suppress PbI_2_ formation [53]. 

Light-induced degradation is also studied by in situ XRD in vacuum. The results show that FACs-perovskite shows better light stability than MA-perovskite [54]. Under light conditions with an Xe lamp at 400 W/m^2^ and after 30 min, the (100) peak of MA-perovskite showed an increased peak width followed by peak splitting at 14.3°, while FACs-perovskite shows no obvious change. For longer light exposure, MA-perovskite further decomposed into metallic lead (Pb_0_) and lead iodide (PbI_2_), as shown by diffraction peaks at 12.7°. Factors other than light could greatly affect the degradation pathway of FACs-perovskite. In vacuum, photodegradation mainly creates iodine vacancies. But in air, the lead salts are formed due to removal of organic parts under the presence of oxygen and water. The reason for this is that FACs-perovskites would form I-element domains under light conditions and thus accumulate strain, which can have a stabilizing effect on the perovskite. 

Synchrotron-based grazing incidence X-ray diffraction (GIXRD) and grazing incidence wide-angle X-ray scattering (GIWAXS) has been widely used in the structural determination of perovskite solar cells with better surface sensitivity [55,56]. It studies the crystallinity of the top surface by using an X-ray with a very shallow angle of incident. The advantages of GIXRD and GIWAXS are (1) the large detection area due to elongated incident X-ray spot from a very shallow angle; (2) the adjustable sensitivity in the z-direction by changing the grazing incidence angle from very shallow ~0.02° (ultrahigh surface sensitivity) to less shallow ~1° (relative bulk sensitive); and (3) the high sensitivity from high X-ray flux and quick angular information in combination with a two-dimensional surface detector (MarCCD). Ex situ GIXRD and GIWAXS have been widely used as a complementary technique to confirm the structural information of perovskite surfaces [4,14,57]. 

**Figure 4:**
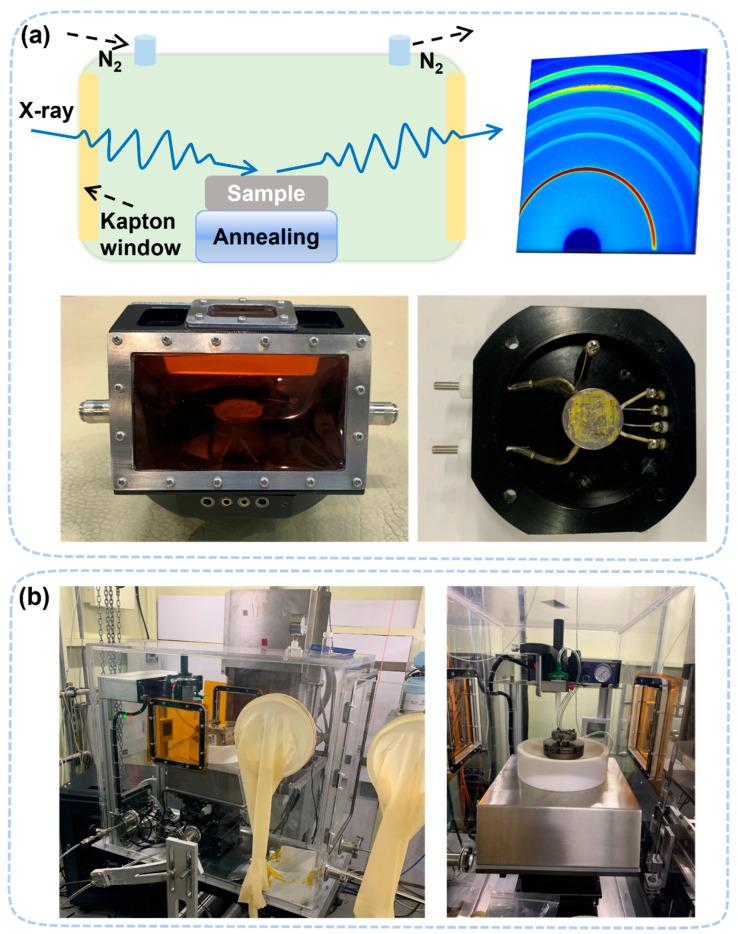
(**a**) In situ heating device with Kapton film windows on both sides. The operating temperature can range from −180 °C (≈liquid nitrogen temperature) to 200 °C. (**b**) In situ glove box, which can be used for in situ spin-coating experiments and in situ degradation experiments, including thermal, humidity, and light decomposition.

To perform in situ/operando GIXRD measurements, the test chamber must adapt to the grazing incidence of the X-ray. A flat top window is not suitable for a very shallow incidence angle (~0.02°). Therefore, the setup must use side widows to perform GIXRD measurements. The typical window materials to seal the chamber is Kapton films, which have good mechanical strength and low X-ray absorption coefficient. Alternatively, a complete glovebox as the test chamber can be used to conduct more complicated studies with stable environmental control, one of which is located in the Shanghai synchrotron radiation facility (SSRF). [58,59,60]. Although this setup is mainly to study the formation of perovskite [61], it can also be used to study the degradation process, as shown in Figure 4. 

In situ GIXRD studies have not only confirmed the formation of hydrate, but it also observed surface-related degradation under various stresses. It confirmed the formation of hydrate and PbI_2_ under a similar relative humidity [45]. Using in situ GIWAXS, Fransishyn et al. showed [46] that the introduction of moisture could increase ion mobility, leading to the screening of the built-in potential and resulting in electrode corrosion. In situ GIWAXS measurements have found that the heat-induced decomposition of the surface of MAPbI_3_ actually starts as low as 80 °C, which is evidenced by the appearance of a PbI_2_-related diffraction ring at ≈0.9 Å^−1^. When the temperature increased from 80 °C to 100 °C, the decomposition progresses faster, showing a degradation time decrease from 1 h to 20 min. The decomposition process, unlike the tetragonal-to-cubic phase transformation, is irreversible. The angle-dependent GIWAXS also revealed that decomposition starts from the surface and progressively advances into the bulk.

### 2.2. Perovskite Degradation Studies by In Situ/Operando Neutron Scattering Techniques

The structural information from degradation can also be studied by neutron scattering. It is a less reported method, mainly due to very few experimental facilities capable of performing it. Neutron scattering can provide considerable and unique structural information, which cannot be obtained using XRD. For example, (i) neutron scattering is capable of studying amorphous phases using a radial distribution function, (ii) a neutron scattering cross-section is less dependent on the atomic number, which is useful to study hydrogen atoms in the crystal structure of perovskite, and (iii) neutrons have a higher penetration depth than lab-based X-ray photons, which enables the study of the structure of large crystals. Right now, there are only two papers reported using an in situ GISANS method [50,62]. To perform such study, an air-tight chamber, like the one used for in situ XRD, is used to study the humidity-induced degradation of MAPbI_3_. Instead of glass, Al was used as the window material because of its low absorption coefficient to neutrons.

**Figure 5:**
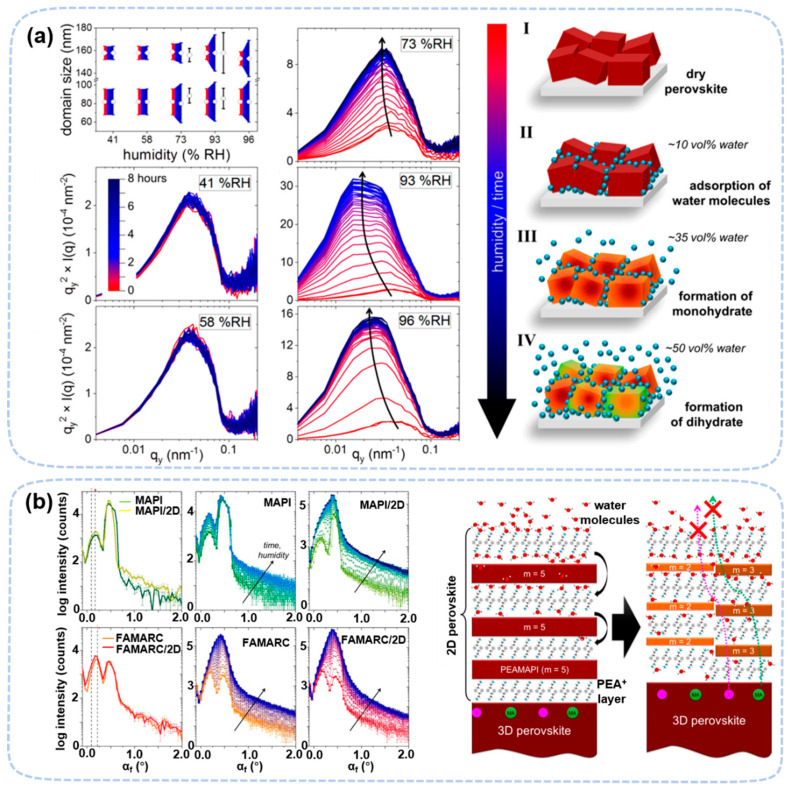
(**a**) Domain sizes for various humidity levels and Kratky-style plots exposed to various humidity levels. The schematic illustration of the degradation pathway [50]. Copyright 2018, American Chemical Society. (**b**) Vertical line cuts of the in situ GISANS measurements, showing the evolution of the perovskite films from the initial state before exposure to 90% RH. The schematic illustration of humidity-induced mechanism in 3D/2D heterojunctions [62]. Copyright 2019, American Chemical Society.

GISANS was used to study water distribution in perovskite. Chlipf et al. determined the formation conditions of hydrates in MAPbI_3_ (Figure 5a) [50]. The RH required for monohydrate is greater than ~70% while the dihydrate requires RH greater than 90%, which are consistent with the in situ XRD studies [48,49,50]. In addition, GISANS can find the RH-dependence of the water content in perovskite as it absorbs on the surface or into the bulk of perovskite depending on the humidity level. Surprisingly, at 41% RH, there is already 10 vol% of water in perovskite. The water content can be as high as 50 vol% at 96% RH. The results also show that water can enter the perovskite without forming a new phase or domain expansion.

GISANS has confirmed that quasi-two-dimensional (3D/2D) perovskite has better moisture stability at a high humidity level [62]. GISANS also revealed that 3D/2D perovskite degrades mainly by phase separation at high humidity levels (~90% RH). The two-dimensional phase with n = 5 (PEA)_2_(MA)_4_PbI_16_ transformed into a more stable n = 2 and n = 3 phase (Figure 5b). The presence of the two-dimensional phase may reduce the migration of ions to the crystal’s surface, such as MA^+^ and I^−^, thus inhibiting the formation of PbI_2_. For the degradation process of 3D/2D perovskite films, the inhibition of ion migration in 2D perovskite may be more important than the hydrophobicity of organic ligands. Although only water-induced degradation was reported in the GISANS study, other types of degradation can also be studied by GISANS because all hybrid perovskites contain hydrogen.

All structural characterization techniques have long probing distance (>1 μm). The operando approach can be easily achieved in a commercial or home-made airtight cell/chamber. However, these setups are not specially design for the study of perovskite and can be further improved by the integration of a heater and/or electrical fields in the cell/chamber to study the structural changes under multiple stresses. For example, a cell with 85 °C and 85% RH condition will be helpful to study degradation under accelerated aging. Table 1 lists most relevant in situ/operando papers on the perovskite film degradation process using crystal structural characterization techniques.

## 3. Composition Characterization Techniques

As hybrid materials, perovskite materials contain multiple elements, which are changed during degradation. Therefore, probing the compositional change at the surface or in the bulk is an important method to study the degradation process. There are many techniques that are sensitive to compositional changes, including thermogravimetric analysis (TGA), mass spectrometry (MS), time-of-flight secondary ion mass spectrometry (ToF-SIMS), photoelectron spectroscopy (PES), and so on.

### 3.1. Perovskite Degradation Studies by Operando Thermogravimetric Analysis Techniques

Thermogravimetric analysis (TGA) measures the weight loss of a sample during heating. This technique has been widely used to study the decomposition mechanism and reaction kinetics of materials. It determines the decomposition temperatures and decomposition kinetics (first order or second order) by weight loss. In perovskite studies, most of the TGA data suggest a first order decomposition process [69,70].

TGA measures the weight change as a function of temperature. Hence, this technique is always working as an operando approach [69,71,72]. Heat-induced MAPbI_3_ decomposition shows two-step mass loss: the first one is the loss of the organic component CH_3_NH_3_I (~25%wt, 220~420 °C) and the second one is the loss of the inorganic PbI_2_ component (~75%wt, above 420 °C). The mass-loss kinetics of the first step has two different loss rates depending on the temperature, suggesting two parallel degradation processes. With the help of other techniques, such as mass spectrometry and Fourier transform infrared spectroscopy, it was determined that these two parallel processes are due to the decomposition of CH_3_NH_3_I through two different routes [69,70,71].

CH_3_NH_3_PbI_3_ (s) = PbI_2_ (s) + HI (g) + CH_3_NH_2_ (g) (1)

CH_3_NH_3_PbI_3_ (s) = PbI_2_ (s) + CH_3_I (g) + NH_3_ (g) (2)

For processes occurring at a lower temperature (~250 °C), Process (1) is dominant. For those occurring at a higher temperature (~300 °C), Process (2) dominates instead. The second process is due to the breaking of the C-N bond, which requires greater energy. The stronger C-N bond associated with the greater enthalpy change can also explain why FA-perovskite has a higher thermal stability than MA-perovskite.

With the exception of heat-induced degradation, TGA has potential in the study of perovskite decomposition combined with other stresses. For example, if water is introduced into the TGA, perovskite materials may appear to have different decomposition kinetics due to synergistic effect of water and heat. The integration of water, light irradiance, and electric fields into the TGA system will probably give new insights into the decomposition kinetics of perovskite. To realize such a goal, a closed cell will be needed to control environmental changes like humidity and light. A proper pump to remove volatile components is required to allow for the determination of mass loss. These modifications can allow TGA to study new perovskite materials, such as quasi-two-dimensional perovskite or passivated perovskite, to directly explore their degradation kinetics under different stresses.

### 3.2. Perovskite Degradation Studies by In Situ Mass Spectrometry and Time-of-Flight Secondary Ion Mass Spectroscopy

**Figure 6:**
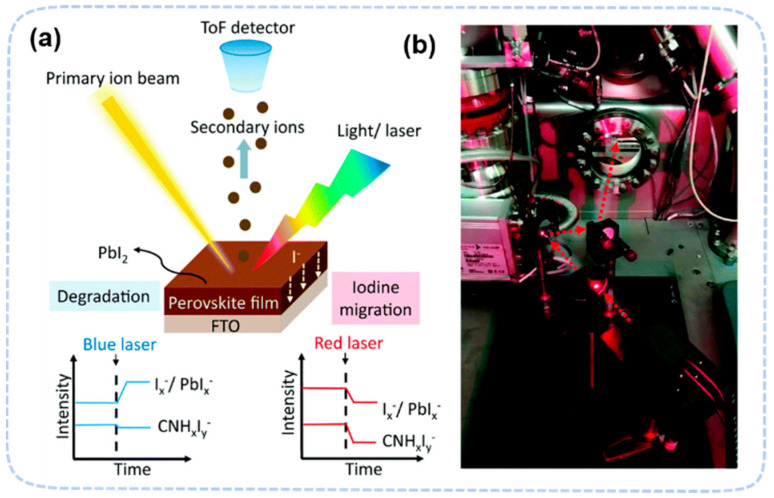
(**a**) Schematic diagram of the experiment with light-induced chemical changes of perovskite films in real time. (**b**) The actual object image corresponding to the experiment [73]. Copyright 2018, the Royal Society of Chemistry.

Mass spectrometry (MS) can identify gaseous components from perovskite degradation by detecting their mass-to-charge ratio. It is an extreme sensitive technique, capable of detecting signals down to 10^−13^ torr, but it also requires a ultrahigh vacuum condition to operate. Therefore, all MS studies are conducted using an in situ approach. With the exception of probing gaseous products, MS is often combined with a sputter gun to probe the solid products on surface. One of the most widely used combination is time-of-flight secondary ion mass spectroscopy (ToF-SIMS), which uses the flying time of secondary ions to determine its mass. ToF-SIMS has a wide detection range of molecular mass and high sensitivity to selected mass. It also has lateral resolutions, which allows it the measure degradation in a single domain. For in situ measurements, ToF-SIMS can integrate external stresses, like illumination, heat, and water, into the system with simple modifications (Figure 6).

**Figure 7:**
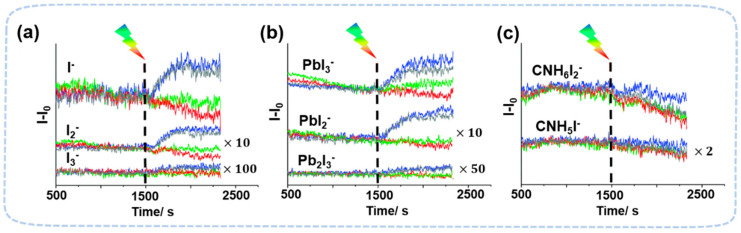
ToF-SIMS signal of chemical components of CH_3_NH_3_PbI_3_ with different light illuminations. (**a**) I^−^, I_2_^−^, and I_3_^−^, (**b**) PbI_3_^−^, PbI_2_^−^ and Pb_2_I_3_^−^, (**c**) CNH_6_I_2_^−^ and CNH_5_I^−^, using a white light, blue laser, green laser, and red laser [73]. Copyright 2018, the Royal Society of Chemistry.

Light-induced degradation in perovskite has a clear wavelength dependence [74,75,76]. The shorter wavelength can break the chemical bonds in perovskite and cause a direct decomposition. In MAPbI_3_ films, MS detected CH_3_NH_2_ and HI under ~395 nm light, which is due to the deprotonation of CH_3_NH_3_^+^ cations [20,76]. The decomposition process is accelerated by presence of oxygen, probably due to the reaction of superoxide and MA. Superoxide is formed by the reaction of molecular oxygen and photogenerated electrons. In a MAPbI_3_-based device, further degradation mechanisms, including (a) CH_3_NH_2_ + HI, (b) NH_3_ + CH_3_I and (c) Pb_0_ + I_2_, were found under prolonged white-light illumination and heating condition (40~80 °C) [75]. In light-induced degradation, the lateral resolution of ToF-SIMS has been used in the cross-section mapping of the surface of perovskite to confirm the wavelength-dependent ion migration. Before illumination, the 2D image of I^−^ ions showed a uniform distribution. After 10 min of red-light irradiation (~635 nm), the irradiated area showed a significant signal decrease. The migration of iodine-related species is quasi-reversible when the wavelength is long (~635 nm) (Figure 7) [73]. At shorter wavelengths (blue light, 470 nm), light induced a more significant degradation of MAPbI_3_ to produce PbI_2_ based on the increase in I_x_^−^ and PbI_x_^−^ signal strength. ToF-SIMS also found that light induces MA^+^ accumulation at the perovskite/PCBM interface and I^−^ accumulation at the perovskite/NiO interface. Such accumulation may be beneficial to the device efficiency because it may change the band structure, leading to a rise in open-circuit voltage and fill factor [77]. With the presence of both heat and light illumination, ToF-SIMS confirmed that degradation is accelerated mainly due to Au diffusion into the perovskite layer [78].

**Figure 8:**
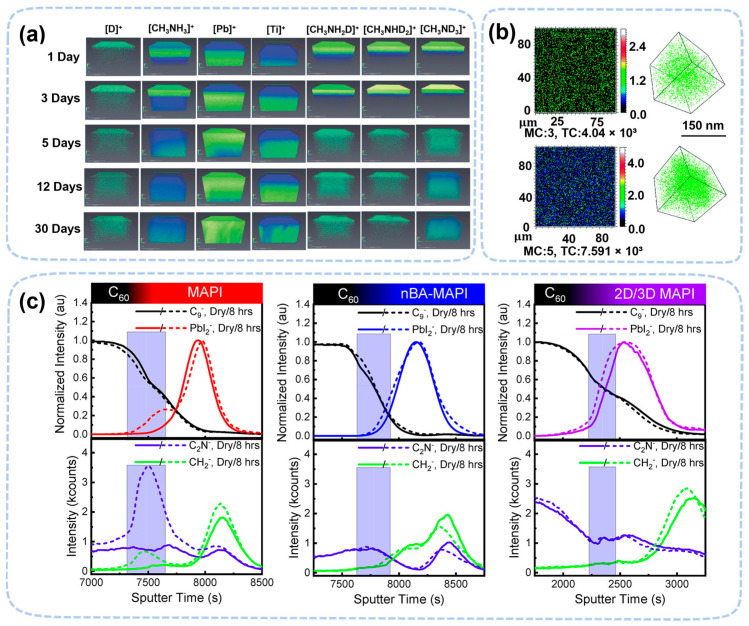
(**a**) Three-dimensional images for deuterium, MA^+^, PbI_2_, and TiO_2_ at different exposure times [79]. Copyright 2017, Wiley. (**b**) ToF-SIMS imaging of D_2_O in thin films at ~150 nm depth and three-dimensional images [81]. Copyright 2017, the Royal Society of Chemistry. (**c**) ToF-SIMS depth profiles of MAPI, nBA-MAPI, and 2D/3D MAPI devices, before and after exposure to humidity [84]. Copyright 2019, American Chemical Society.

In situ ToF-SIMS confirms water penetration in the low-water-exposure condition (~20% RH) even earlier than the condition revealed by GISANS [50]. By using deuterated water (D_2_O), TOF-SIMS also revealed a hydrogen exchange from the adsorbed water. Lin et al. found both methylamine vapor emission and PbI_2_ formation during D_2_O penetration [79], indicating that the surface of MAPbI_3_ might undergo decomposition in the very-low-water-exposure condition. The hydrogen exchange was confirmed by the signal of CH_3_NH_2_D, CH_3_NHD_2_, and CH_3_ND_3_ in their TOF-SIMS data. (Figure 8a) [80]. ToF-SIMS also confirmed that water could penetrate deep into the MAPbI_3_ crystal, but not on the surface, in a humid environment (~85% RH) (Figure 8b). [81]. Intriguingly, ToF-SIMS observed the penetration of oxygen into the perovskite film, which explained the synergistic effect of water and oxygen during degradation [82,83]. TOF-SIMS confirmed that 2D (nBA-MAPbI), and 2D/3D perovskite was much more stable than MAPbI_3_ [84]. The PbI_2_ peak broadened at the interface of C_60_ and perovskite, while the peak was unchanged in 2D and 2D/3D perovskite. The change in this peak may be related to the hydrate of perovskite or PbI_2_. The content of water in the 2D and 2D/3D perovskite is less due to the blocking effect of the 2D cation (Figure 8c).

**Figure 9:**
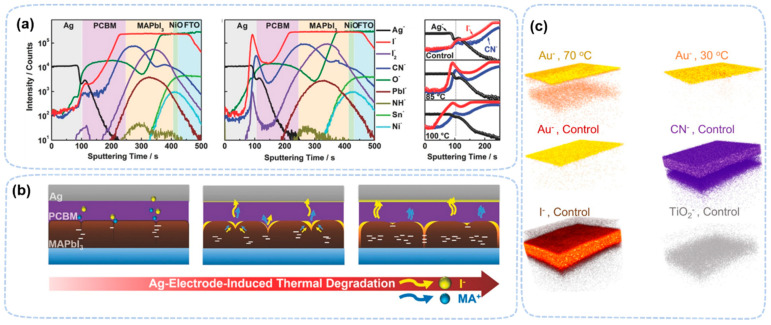
(**a**) ToF-SIMS depth profiles before and after heat treatment at 85 °C for 24 h. (**b**) The degradation mechanism of Ag-electrode-induced thermal degradation [85]. Copyright 2017, Wiley. (**c**) Three-dimensional images of the aged perovskite devices with Au electrode at 30 °C and 70 °C [34]. Copyright 2016, American Chemical Society.

ToF-SIMS was also used to study ion diffusion in perovskite related interfaces. At an elevated temperature, iodine ions diffuse through the PCBM layer and react with an silver electrode in a FTO/NiO/MAPbI_3_/PCBM/Ag device, as was found in a depth study [85] (Figure 9a,b). A similar diffusion was observed with Au (top electrode) (Figure 9c) [34]. A temperature gradient may accelerate the ion diffusion, which shows an obvious difference between uniform heating and non-uniform heating [86]. The nonuniform heating is more severe in perovskite light-emitting diode (LED) devices due to its large driving current, leading to a very short lifetime compared to that of PV devices. In LED-aged devices, ToF-SIMS confirms the severe ion migration of Br ions [87].

**Figure 10:**
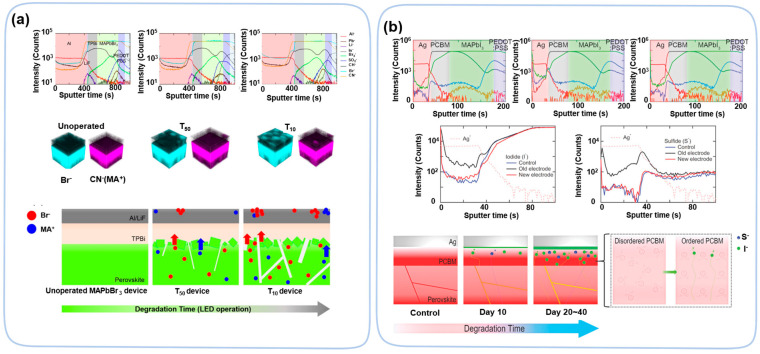
(**a**) ToF-SIMS depth profiles of pristine, the half-lifetime of luminance (T_50_) and the 10% lifetime (T_10_) of luminance devices, respectively. Three-dimensional images of Br^−^ and MA^+^ distribution. The degradation mechanism of the PeLED caused by ion migration [89]. Copyright 2019, American Chemical Society. (**b**) The degradation mechanism of MAPbI_3_ perovskite solar cells as a function of the degradation time [91]. Copyright 2018, Wiley.

Ion migration caused by electric fields is another topic for TOF-SIMS studies. There are two types of ion migration in perovskite devices: perovskite-related ions moves towards HTL and ETL and metal atom diffusion into the ETL/HTL or even perovskite layer [88]. ToF-SIMS 3D mapping demonstrates that the concentration of Br^−^ and MA^+^ on an Al electrode increases with the time of operation (3~30 min) of the LEDs, confirming that the degradation of the device is due to the migration of MA^+^ and Br^−^ towards the ETL direction (Figure 10a) [89]. ToF-SIMS observed ion migration in both regular and inverted PV devices [90,91]. A difference in the overlap of MA^+^ and HTL distributions at the interface confirms the ion migration from the perovskite layer to the HTL after device operation. The similar migration of negative ions is also observed at perovskite/ETL interface [90]. In inverted structure devices, negative ions (I^−^) diffused from the perovskite layer and HTL to the ETL (PCBM), which caused the deterioration of the device’s efficiency (Figure 10b) [91]. In electric field studies, TOF-SIMS found that Au atoms diffuse into perovskite layer, causing the device performance to drop to low efficiency [92,93,94]. Thus, ToF-SIMS is a powerful tool to determine ion migration in perovskite materials.

Both MS and ToF-SIMS can be further integrated with heating, electrical field, light irradiance, and gas exposure (water, oxygen, etc.). Although it is difficult to reach very high partial pressure of gases, its accuracy and sensitivity make these techniques very suitable to study the degradation mechanism of perovskite materials.

### 3.3. Perovskite Degradation Studies by In Situ/Operando Photoelectron Spectroscopy Techniques

Photoelectron spectroscopy (PES) is a surface-sensitive technique aiming to measure the electronic structure and valance states of elements. Because it uses electrons as its major detection particle, PES usually requires a high vacuum to operate, though a rough vacuum and even atmospheric condition can still be used in specially designed systems. PES is widely used by both ex situ and in situ studies. PES can be divided into X-ray photoelectron spectroscopy (XPS) and ultraviolet photoelectron spectroscopy (UPS). The main difference between the two techniques is the photon source. XPS, with a higher photon energy (Al/Mg Kα, 1486.7 eV/1253.7 eV, respectively), is capable of measuring the binding energy of electrons in core levels, determining the types and chemical shifts of elements. UPS, with a lower photon energy (typically 21.2 eV from He I), is suitable to measure the valance electrons and the region close to Fermi level. Ex situ PES confirmed surface compositional changes under external stresses, such as the gradual loss of CH_3_NH_3_^+^ and I^−^ from MAPbI_3_ in air [95]. Eventually the perovskite turns into various Pb compounds, including Pb(OH)_2_, PbO, PbCO_3_, depending on the decomposition conditions [96].

In situ PES studies require the integration of one or multiple degradation stresses into a high vacuum environment. Water and oxygen are introduced directly into the vacuum chamber through leakage valves [97]. However, the partial pressure of both substances cannot exceed the minimum requirement of the vacuum system (typically 10^−5^ to 10^−4^ mbar). The light irradiation is introduced through the viewport of the vacuum chamber [59,98]. Most PES manipulators are integrated with a heater, allowing for in situ heating. Only the in situ electrical field is difficult because the top electrode may block photoelectron signals and the electrical field interferes the kinetic energy measurement of photoelectrons.

**Figure 11:**
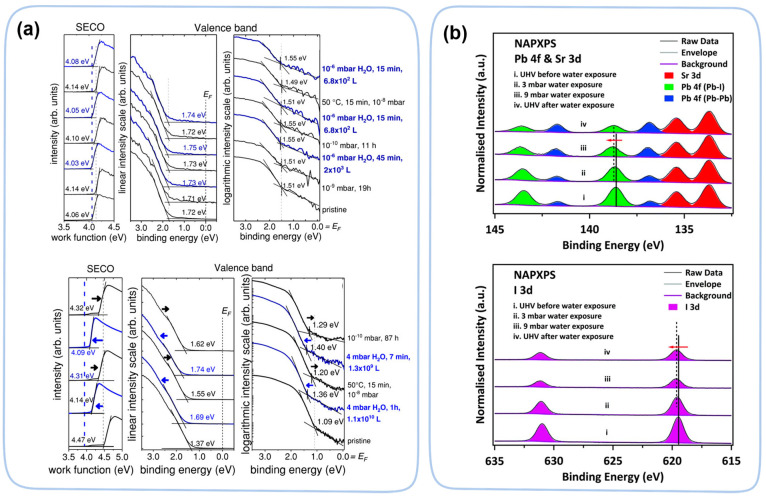
(**a**) UPS spectra of MAPbI_3−x_Cl_x_ perovskite films under different H_2_O exposure with 10^−6^ mbar and 4 mbar [99]. Copyright 2018, Wiley. (**b**) NAPXPS spectra of perovskite materials at core level of Pb 4f and I 3d under various water exposure conditions with 3 mbar and 9 mbar [100]. Copyright 2017, the Royal Society of Chemistry.

In situ PES studies revealed subtle change in electronic structure when perovskite is exposed to water. At low water pressure (10^−6^ mbar), water is adsorbed onto the surface of the solution-processed perovskite film MAPbI_3−x_Cl_x_, which decreases its work function (WF) by inducing surface dipoles (Figure 11a) [99]. This value of WF is fully reversible after annealing (~50 °C) in a ultrahigh vacuum, suggesting fully reversible WF changes without any composition degradation of the perovskite. However, at a higher water pressure (4 mbar), the change is beyond the reversible range. Under high water pressure conditions (10^10^ Langmuir), an electron transfer from adsorbed water to MAPbI_3_ is observed, resulting in n-type doping [97]. These studies revealed electronic structural changes in perovskite before the intermediate phase or decomposition. From in situ PES studies, perovskite materials are less sensitive to the presence of oxygen [97,99]. Only under the very high partial pressure of oxygen, a small WF change is observed on the surface of the perovskite, probably due to a charge transfer of the surface-absorbed oxygen atoms.

Although normal in situ PES cannot be performed at very high water partial pressures, a commercially available modification (near-ambient-pressure XPS (NAP-XPS)) can extend the in situ water study to near ambient condition. In NAP-XPS, a sample is sealed in a chamber with an extremely small exit (300 μm) to collect emitted photoelectrons. Multistage differential pumping is used reduce the pressure at least 10-million-fold (1) to assure a long enough mean-free path for the photoelectron to reach the analyzer and (2) to keep low enough pressure in the analyzer side to maintain its working condition. Using NAP-XPS, water-induced degradation shows dependence by its formation methods. Ke et al. found that MAPbI_3_ prepared by thermal deposition decomposed into PbI_2_, HI, and NH_3_ at 9 mbar of water partial pressure (Figure 11b) [100], while Chen et al. reported that both MAPbI_3_ and FAPbI_3_ prepared by spin coating do not decompose at up to 24 mbar of water partial pressure [101].

Various wavelengths of light will cause different decomposition processes [59,102]. With UV light at 366 nm wavelength or shorter, perovskite materials undergo rapid photodisintegration. It causes the ion migration of A-site cations and the decomposition of the PbI_6_^4−^ octahedral backbone to form Pb_0_. When the wavelength of light is longer than (435 nm), the decomposition is dominated by the ion migration of A-site cations. The decomposition under visible light is slow.

The atomic ratio of Cs/Pb changes from 16% to 9% after exposure to 12 h of red light (624 nm), which mainly induces a change in vacancy density. In mixed perovskite containing MA, FA, and Cs, the Pb_0_ signal is the smallest under visible light due to a smaller lattice, limiting the ion migration [103]. Beyond 3D perovskite, mixed perovskite films ((FAPbI_3_)_0.85_(MAPbI_3_)_0.15_) show the formation of Pb_0_, which can be partially reversed by the photoelectron-transfer-induced oxidation of Pb_0_ [104]. Therefore, in situ PES can investigate the compositional and valance state changes that occur during perovskite degradation.

In perovskite degradation studies, a few general modifications can further improve the capability of in situ XPS. For water and oxygen exposure, modifications like NAP-XPS change its operation from in situ to operando. For light irradiance, a proper window material needs to be chosen if a short wavelength is used. Due to the reduced transmittance to wavelengths over 400 nm of corning 7056 glass or Kodial glass, fused silica is a better choice as more than 90% of the transmittance is to a wavelength that is less than 300 nm. Due to the constraint of chamber itself, the light intensity needs to be carefully measured either by in-advance measurement when the chamber is exposed to air or by a calibrated sample, which gives a linear response in photocurrents. Due to limited heat passivation in vacuum, proper cooling should be considered to avoid an unwanted heating effect when light intensity is high. Although NAP-XPS is a power modification to achieve operando operation, a two-chamber system to separate water exposure and XPS measurement is an alternative choice. This is because the perovskite degrades relatively slow, so a stepwise degradation measurement could easily provide very high water partial pressure without losing too much information in an operando measurement. We believe that an operando approach in PES is important for future study of perovskite degradation. Table 2 lists most relevant in situ/operando papers on the perovskite film degradation process using these techniques.

## 4. Morphology Structure Characterization Techniques

Since degradation will eventually change the morphology of perovskite films, it is one of the most straightforward indications of the degradation process. In devices, the film morphology is even more critical because morphological changes directly damage the device’s integrity and results in the premature failure of the device. For example, perovskite undergoes recrystallization due to hydration and dihydration, causing morphology changes. This change may not decompose the perovskite film, but it threatens the film’s continuity. Therefore, more efforts should be devoted to the morphological study of perovskite degradation.

Many techniques can probe the morphological changes that occur during degradation. Some of these technologies may require vacuum conditions, including electron microscopy (EM) (scanning electron microscopy (SEM) and transmission electron microscopy (TEM)). Others may work in atmospheric condition, such as scanning probe microscopy (SPM) and optical microscopes.

### 4.1. Perovskite Degradation Studies by In Situ/Operando Electron Microscopy Techniques

SEM is the most widely used technique to characterize the morphology of the surface of perovskite. It easily distinguishes submicrometer domains on perovskite surfaces. TEM has higher spatial resolution and is often used to identify atomic crystallinity in perovskite. Ex situ SEM and TEM have been used to characterize morphological changes under various of stresses. Among these studies, water-induced morphological changes in MAPbI_3_ have been thoroughly studied [43,96].

**Figure 12:**
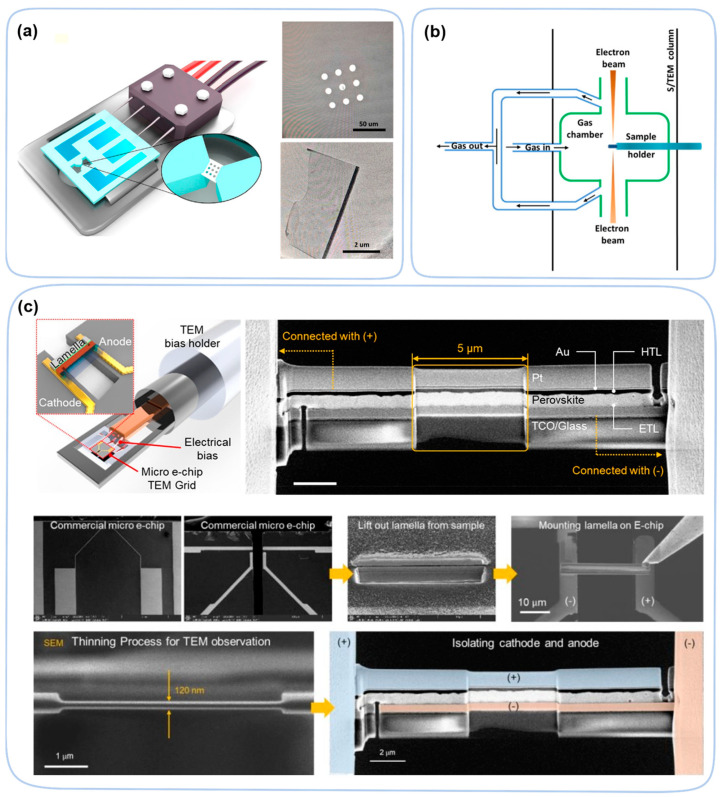
(**a**) Schematic illustration of the heated sample holder inserted in the in situ S/TEM chamber [105]. Copyright 2020, Elsevier. (**b**) Schematic illustration of the in situ S/TEM device with heating function as well as gas pathway through the SiN_x_ window of the confined chamber [106]. Copyright 2016, American Chemical Society. (**c**) Schematic illustration of the electrical bias holder of in situ TEM. The nano perovskite sample was mounted on the micro e-chip during the FIB manufacturing process [107]. Copyright 2021, American Chemical Society.

Similar to PES, SEM and TEM techniques require a ultrahigh vacuum and are compatible with in situ approaches. Modifications to provide heating/light/electrical biases to samples are commercially available, depending on the model of SEM and TEM. Heating and bias functions can be achieved as a standard add-on of the sample holder. A typical heater on a sample holder can reach 1300 °C, which is more than sufficient for perovskite studies (Figure 12a) [105]. Light illumination can be simply integrated by using a proper viewport with high transmittance to the wavelength of interest. To expose perovskite to water or other gaseous stresses, a gas holder is needed as well as a hermetically sealed reaction chamber for TEM. To keep high electron transparency, the material of the window of the reaction chamber must be very thin (10 nm), which can be achieved by using a material such as silicon nitride (SiN_x_). Due to fragility of the window, the pressure of the reaction chamber must be carefully controlled (Figure 12b) [106]. The bias function on a sample holder can reach 100 V DC or AC, which is sufficient to apply a lateral electrical field of 10^8^ V/m between two electrodes separated by 1 μm. To facilitate the observation of the perovskite layer by in situ TEM, the solar cell device needs to be thinned by a focused ion beam (FIB), leaving only the perovskite layer (thinned to 300 nm) [107]. Then, the entire circuit is conducted by vapor mental Pt to ensure its conduction. Device electrodes (Au and ITO) need to be cut using FIB to prevent device shorting and enable electrical biasing (Figure 12c). In addition to characterize the degradation process, in situ techniques are also used in the perovskite crystallization and formation processes [108,109].

Most of the water-induced morphological changes are observed by ex situ SEM [43,101]. At low RH levels (~60%) or a short exposure time (a few hours), the domain size gradually changed, indicating the recrystallization of perovskite films. When RH level is 80% or higher, large voids appear on the perovskite’s surface, probably due to localized water accumulation on the surface of the perovskite. These large voids may severely damage device integrity and need to be investigated further. However, due to the difficulty of introducing high enough water partial pressure into in situ SEM or TEM, in situ/operando studies of morphological changes at high humidity levels may need to rely on techniques without a vacuum.

**Figure 13:**
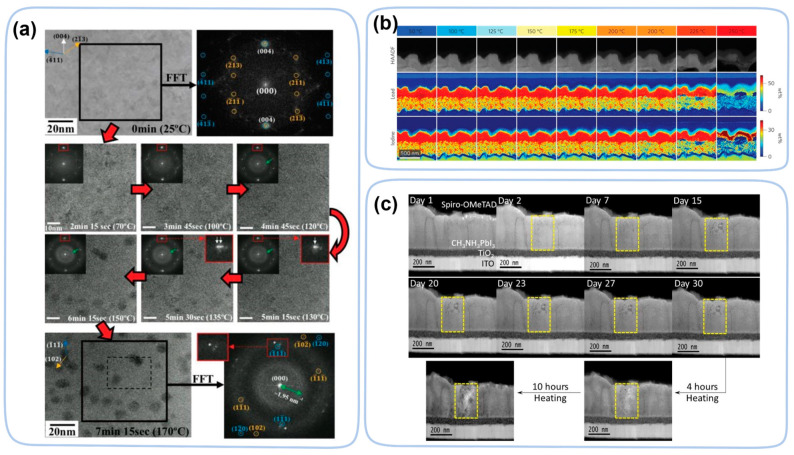
(**a**) HRTEM images and FFTs showing the detailed thermal degradation procedure from 25 to 170 °C [110]. Copyright 2018, Wiley. (**b**) HAADF images and EDX elemental maps for iodine and lead acquired after heating at different temperatures [111]. Copyright 2016, Springer Nature. (**c**) Real-time cross-sectional HAADF images of a typical perovskite solar cell with different heating times [112]. Copyright 2016, American Chemical Society.

In heat-induced degradation, in situ TEM witnessed crystallinity changes at a much lower temperature in MAPbI_3_ [110]. The crystalline MAPbI_3_ turned into its amorphous phase at temperatures as low as 130 °C before it decomposed into PbI_2_ (150 °C) (Figure 13a). The decomposition temperature of the spin coating method is higher than that of the evaporation method. Due to poorer crystallinity, trigonal PbI_2_ is observed on perovskite by chemical vapor deposition at temperatures as low as 85 °C [35]. Combined with theoretical calculations, it was found that perovskite decomposes in a layer-by-layer manner.

In devices, heat-induced degradation is even more complicated due to interactions between different layers. During the heating process, morphological changes occur at 150 °C, forming holes near the TiO_2_ layer. The I^−^ ions first migrate from the perovskite layer into the HTL, and as the temperature increases (~175 °C), the Pb atoms migrate from perovskite layer towards the Au electrode (Figure 13b) [111]. Under the heating conditions, the perovskite layer decomposes and forms PbI_2_, and in addition, holes are formed. Instead of heating via the sample holder, the heating can also be partially achieved by parasite heating from a halogen lamp. In contrast, the decomposition of the perovskite is faster using the latter method. Decomposition occurs at temperatures as low as 50–60 °C (Figure 13c) [112]. Such accelerated decomposition is probably due to synergistic effect of heating and light illumination.

**Figure 14:**
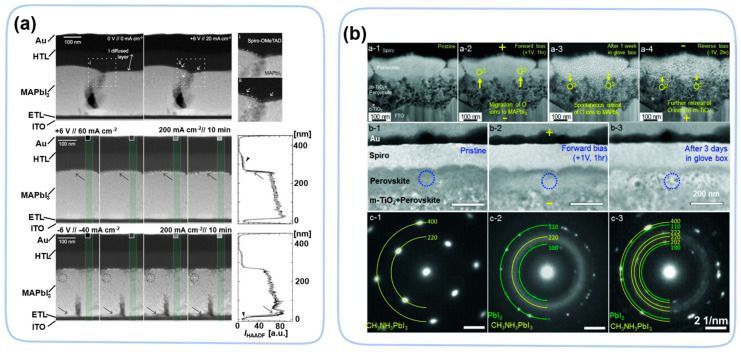
(**a**) STEM HAADF micrographs showing changes in morphology of the MAPbI_3_ layer with +6 V applied to the HTL [116]. Copyright 2016, American Chemical Society. (**b**) Effects of in situ electrical biasing on morphology and structure of MAPbI_3_ [117]. Copyright 2018, Wiley.

In an electric field, ion migration may also affect morphology of device [113,114,115]. An operando cross-section TEM on the cell structure of perovskite material, ITO/PCBM-polyetheylenimine(ETL)/MAPbI_3_/spiro-OMeTAD(HTL)/Au, observed PbI_2_ domain formation due to ion migration driven by an electric field [116]. Under +6 V positive bias, the anions migrated toward the HTL layer and caused formation of PbI_2_ nanoparticles at the MAPbI_3_-HTL interface (Figure 14a) (i.e., the positive direction of the electric field). Inversely, voids can form at the interface between the perovskite layer and the ETL layer. Except for the migration of ions in the perovskite layer, atoms and ions in the ETL/HTL layers may also migrate and degrade the perovskite layer. In a device (FTO/compact-TiO_2_/mesoporous-TiO_2_/MAPbI_3_/Spiro-OMeTAD/Au) [117], oxygen ions migrate from the ETL layer to the perovskite layer, which leads to the formation of amorphous MAPbI_3_ and PbI_2_ (Figure 14b) under forward bias of only +1 V. However, this morphological change is partially reversible under a reverse bias of −1 V. In addition, illumination causes the photodegradation of perovskite materials, forming nanoparticles Pb_0_ at the grain boundaries [20]. Through in situ TEM, the perovskite material first forms the core–shell structure intermediate Pb@PbI_2−x_ and then decomposes into Pb_0_ [118].

Most in situ studies are performed using TEM. However, if similar modifications are applied to SEM, in situ SEM will be an alternative method to studying degradation as well. To expend the capability of in situ approaches, the integration of multiple functions on the sample holder to provide heating, electrical fields, water exposure, and light illumination should be considered. In situ EM is complementary in many ways to the other techniques, such as X-ray diffraction, ToF-SIMS, PES, and so on.

### 4.2. Perovskite Degradation Studies by Operando Atomic Force Microscopy Techniques

Atomic force microscopy (AFM) does not require a vacuum to operate. Except for surface tomography, AFM can be used to study the mechanical properties and ferroelectricity of perovskite films. The only shortcoming of AFM is its slow scanning speed, which takes much longer to obtain images from the sample’s surface. Due to needed fewer requirements to operate, AFM has great potential to work in both in situ and operando approaches. Conductive atomic force microscopy (C-AFM) and Kelvin probe force microscopy (KPFM) can be used to measure the current-voltage curves of samples to determine the I–V parameters of perovskite PV devices, such as *V*_OC_ and *J*_SC_ [119,120].

In situ AFM also need a closed cell to control the local environment. Currently, in situ AFM studies use such a cell to control humidity. Other stresses, such as light illumination and electrical fields, should be readily available for most commercial AFM systems. The function of heating needs to be carefully managed to avoid severe drifting of AFM tips.

In water-induced degradation, in situ AFM observed no significant morphological changes below ~50% RH. [49,121]. When exposed to above 80% RH, the surface of MAPbI_3_ became rough, indicating the recrystallization of domains. When a single crystalline MAPbI_3_ sample was exposed to the same conditions, the domain shape change was much slower, suggesting that water primarily penetrated along the grain boundaries. [121,122].

For 2D/3D hybrid perovskites with slower degradation processes, an operando study based on AFM might be a better choice. For electron microscopes (SEM and TEM), introducing water into them is always a difficult and risky maneuver, with a longer exposure time pushing such a risk to unacceptable levels. Therefore, future in situ morphological studies should be focused on AFM methods. Table 3 lists the most relevant in situ/operando papers on the perovskite film degradation process using electron microscopy techniques.

## 5. Optical Techniques

Since perovskite materials are mainly used in optoelectronic applications, the decay of performance may directly relate to the changes of its optoelectronic properties, not the structural and morphological changes. There are many techniques that directly characterize the optoelectronic properties of halide perovskite during its degradation, including steady-state photoluminescence (PL), time-resolved photoluminescence (TRPL), absorption spectroscopy, transient absorption spectroscopy (TA), confocal laser scanning microscopy (CLSM), confocal microscopy and laser-beam-induced current (IBIC) mapping, etc. Optoelectronic properties are closely related to the defects in perovskite materials. Therefore, these techniques mainly investigate the degradation related to defect formation in halide perovskites. Most of these optoelectronic techniques use a light-in and light-out strategy during measurement. When combined with a microscope, optical methods have certain spatial resolution to allow for the simultaneous measurement of optical and morphology information. The high penetration depth of light also makes them less difficult to adopt into in situ or operando approaches [123,124].

**Figure 15:**
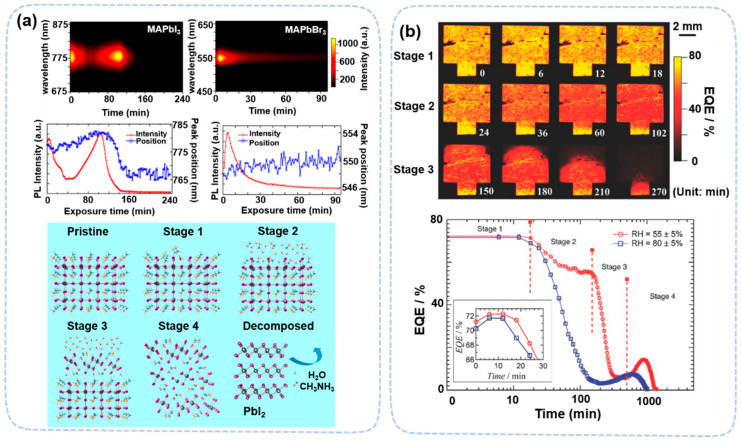
(**a**) PL evolution images and PL emission peak intensity and position of MAPbI_3_ and MAPbBr_3_ exposed to 80% RH as a function of time. Schematic illustration of the evolution of MAPbI_3_ crystal structure under water exposure. Reproduced with permission [123]. Copyright 2018, American Chemical Society. (**b**) EQE maps of a complete perovskite solar cell device after exposure to ~50% RH. Reproduced with permission [125]. Copyright 2016, Wiley.

In humidity-related degradation, in situ PL and TRPL measurement revealed the multistage degradation of MAPbI_3_ and MAPbBr_3_ under 80% RH, determined by exposure time. From PL peak evolution, MAPbI_3_ undergoes a four-stage degradation process, consisting of surface passivation, free-electron doping, interfacial hydration, and bulk hydration (Figure 15a). Among them, the first two steps are defect-related [123]. In the first stage, PL signals increase due to the suppression of surface defects by water adsorption (0~4 min). In the second stage, water molecules penetrate the perovskite film and act as an electron donor and quench PL signals (4~50 min). The last two stages are related to monohydrate (60~100 min) and dihydrate (100~240 min). In contrast, MAPbBr_3_ does not form hydrates under the same condition. This only occurs in the first two stages. A similar degradation process in a complete device is confirmed by Song et al. using in situ laser-beam-induced current (IBIC) imaging [125]. This process can occur at lower humidity conditions (~55% RH), which is probably due to interfacial interactions (Figure 15b).

It is interesting that 2D perovskite appears to be less photostable than 3D perovskite [20,36]. This is due to the presence of more surface Pb-I bonds (along the c-axis plane) in 2D perovskite materials than those of 3D perovskite, which is undercoordinated and less stable under light conditions. In situ CLSM studies found that 2D perovskite turns into PbI_2_ under blue-laser illumination conditions (488 nm, 120 mW cm^−2^) [36]. Table 4 lists the most relevant in situ/operando papers on the perovskite film degradation process using optical techniques.

## 6. Conclusions and Outlook

### 6.1. Conclusions

With the help of in situ or operando approaches, understanding of the perovskite degradation process has been significantly advanced. From these studies, the degradation of perovskite is a complex process that is sensitive to several types and strengths of external stresses. There is also clear evidence that multiple stresses have a synergistic effect on perovskite degradation. The synergistic effect on degradation has been observed between water and heat, light and heat, and probably a general effect between different stresses [106,112]. Degradation may also be affected by preparation methods and the composition of the perovskite as well.

From the in situ and operando studies, the degradation of perovskite under each stress can be systematically summarized: (1) Water-induced degradation is the most complex, inducing multiple stages of changes in perovskite materials. At a low humidity of 10^−6^ mbar, MAPbI_3_ undergoes defect passivation followed by water-induced n-type doping. When the humidity level increases (~20% RH), water penetrates the perovskite crystal and grain boundaries without causing additional changes. When the humidity level reaches ~80% RH or higher, the perovskite forms intermediate states (monohydrate and dihydrate compounds) and eventually decomposes to PbI_2_, depending on the exposure time. (2) Heat decompose perovskite directly. For example, MAPbI_3_ decomposes into PbI_2_ at about 300 °C, and the gaseous product may further decompose into NH_3_ and CH_3_I, depending on the temperature. Though this temperature is much higher than normal operational temperature (~60 °C), decomposition happens much earlier on surface at temperatures as low as 85 °C in chemical-vapor-evaporated MAPbI_3_ samples. The degradation may be accelerated when O_2_ or sunlight is present at the same time. (3) Light-induced degradation depends on the wavelength used. When the wavelength is shorter than ~430 nm, light can the break bonds in organic cations and quickly turn MAPbI_3_ into PbI_2_. When the wavelength is longer, the damage to the perovskite is much slower. The degradation is mainly caused by the photo-induced electric field, causing ion migration and phase separation. (4) Ion migration and phase separation caused by electric field slowly decompose perovskite into inert products like PbI_2_. The migration of ions is determined by the strength and direction of the electric field. The migration of ions has many possibilities, including the migration of anions to the HTL, migration of cations to the ETL, HTL and ETL penetrating the electrode, electrode atom/ion migrating to perovskite interfaces and so on.

### 6.2. Outlook

After a decade of research, many kinds of modifications have been applied to perovskite materials to achieve higher performance and stability, including composition engineering (e.g., single cation to multiple cations) [127,128,129], surface engineering/passivation [130], and preparation method optimization [131,132]. The most stable device reported today has exceed 10,000 h of operation [133]. With improved stability, the investigation into its degradation has also become increasingly challenging. Therefore, to keep the pace of the improvements to its stability and to study the determinate pathway in these perovskite materials, improvement of in situ and operando approaches of characterization techniques is also urgently needed. To achieve this goal, we suggest following directions on the future improvements:

(1) Combine multiple degradation factors in studies.

Most of the current studies use only one degradation stress for simplicity. The studies that use two or more stresses noticed very different degradation processes, probably due to synergistic effects. Therefore, to investigate degradation under multiple stresses would be more important for devices. The integration of multiple stresses into experimental setups is a viable way to improve the capability of the techniques used.

(2) Extend the measurement range of in situ and operando techniques (higher humidity, higher temperature, and so on).

Due to the intrinsic constraints of certain techniques, in situ and operando techniques have limited operational range. For example, for vacuum-related techniques, like MS, SIMS, PES, SEM, and TEM, it is difficult to achieve realistic humidity levels in in situ studies. However, many degradation processes are intensity-dependent. For instance, a WF change caused by water-adsorption-induced degradation starts at 10^−6^ mbar [99], while water penetration through the grain boundary starts at RH levels up to ~20%, and water-induced recrystallization needs 80% RH or higher [121]. Therefore, a wider range is crucial for studying perovskite degradation in detail. Furthermore, if the range is beyond normal operational conditions, accelerated degradation can be achieved to greatly save experimental time. Currently, the double 85 test protocol (85 °C and 85% RH) has been widely used in PV devices, but this condition has not been used to study the degradation mechanism of perovskite devices.

There are a few guidelines to extend the range of in situ and operando studies. For techniques working at atmospheric condition, such as XRD or AFM, a closed chamber is commonly used to control stresses. Therefore, the modification will be mainly on how to extend the working range of the chamber itself. The chamber should be designed to withstand the stress properly, such as inert water and water corrosion resistance (for higher water partial pressure), heat resistance (for higher temperature), transparency (for higher light illumination), and electrical insulation (for higher electric fields). For techniques requiring a vacuum to operate, there are two strategies to extend the range for pressure. The first strategy is to design a specialized setup to withstand the huge pressure difference, such as NAP-XPS, which uses differential pumping to withstand a pressure difference of more than 8~9 magnitudes. Such a strategy is also used in SEM and TEM to design a proper window material to be electron-transparent and withstand large pressure differences, such as graphene. Another strategy is to separate the measurement and exposure step, making the whole process pseudo-operando. Due to slower degradation of perovskite, by using this strategy, realistic conditions are much simpler to achieve without sophisticated modification to the current setup.

(3) Integrate multiple techniques.

Since perovskite degradation is a complex process, including structural, compositional, morphological, and electronic changes, it is important to study it from multiple aspects simultaneously. Integrating multiple characterization techniques can greatly enhance our understanding of the crucial steps in the degradation process and how they affect the device’s performance and lifetime. Some of the techniques can be easily integrated, such as PES and MS, which can detect compositional changes from solid and gaseous products simultaneously, giving a full picture of decomposition process. Similarly, vacuum-based techniques, like SEM, may easily combine with optical techniques to investigate the relation morphological changes and ion migration.

With the proper modification of current in situ and operando techniques, we believe that the degradation of perovskite can be fully understood and that the stability of this material can be further improved to fulfill commercial requirements.

## Figures and Tables

**Table 1:** Summary of in situ/operando techniques applied in crystal structure studies.

Perovskite Films	Exposure Condition	Techniques	Ref.
MAPbI_3_	Water (27~90% RH)	XRD	[32]
MAPbI_3_	Water (80% RH)	XRD	[48]
MAPbI_3_	Water (80% RH)	XRD	[49]
MAPbI_3_	Water (65% RH)	XRD	[47]
CsFAPb(IBr)_3_	Heat (80~320 °C)	XRD	[51]
MAPbI_3−x_Cl_x_	Heat (28~400 °C)	XRD	[53]
FACsPb(IBr)_3_, MAPb(IBr)_3_	Light	XRD	[54]
3D/2D	Water (90% RH)	XRD	[62]
MAPbI_3_	Liquid water	XRD	[63]
MAPbX_3_(X = I, Br, Cl)	Heat (30~320 °C)	XRD	[64]
MAPbI_3_	Vacuum/light	XRD	[65]
(CsFAMA)Pb(IBr)_3_	Light and bias	XRD	[66]
MAPb(IBr)_3_	Light	XRD	[67]
MAPbI_3_	Water (80% RH)	GIXRD	[45]
MAPbI_3_	Water (85% RH)	GIWAXS	[46]
MAPbI_3_	Heat (25~130 °C)	GIWAXS	[68]
MAPbI_3_	D_2_O (73% and 93% RH)	GISANS	[50]
3D/2D	Water (90% RH)	GISANS	[62]

**Table 2:** Summary of in situ/operando techniques applied in elemental composition studies.

Perovskite Films	Exposure Condition	Techniques	Ref.
MAPbI_3_	Heat (20~600 °C)	TGA, MS	[69]
FAPbI_3_, MAPbI_3_	Heat (25~700 °C)	TGA, MS	[70]
MAPbI_3_	Heat (30~390 °C)	TGA	[71]
MAPbI_3_	Light (5 × 10^17^ s^−1^ cm^−2^), (blue and UV LEDs)	MS	[74]
MAPbI_3_	Light and heat (white LED/80 mW·cm^−2^, blue LED/71 mW·cm^−2^, Xe lamp/55 mW·cm^−2^; 35~80 °C)	MS	[75]
MAPbI_3_	Light (100 mW·cm^−2^)	MS	[76]
MAPbI_3_	Light (white LED/55 mW·cm^−2^, red laser 80 mW·cm^−2^)	ToF-SIMS	[73]
MAPbI_3_	Light (AM 1.5, 100 mW·cm^−2^)	ToF-SIMS	[77]
Rb_0.03_Cs_0.07_FA_0.765_MA_0.135_PbI_2.55_Br_0.45_	Heat and light (50~70 °C; white LED illumination at 1 Sun intensity)	ToF-SIMS	[78]
MAPbI_3_	84% RH	ToF-SIMS	[79]
MAPbI_3_	Water (25% RH and 85% RH) and O_2_	ToF-SIMS	[81]
MAPbI_3_	O_2_	ToF-SIMS	[82]
MAPbI_3_	O_2_	ToF-SIMS	[83]
MAPbI_3_, nBA-MAPbI_3_, 2D/3D MAPbI_3_	Water (D_2_O, 15 Torr)	ToF-SIMS	[84]
MAPbI_3_	Heat (85 °C and 100 °C)	ToF-SIMS	[85]
FA_0.83_MA_0.17_Pb(I_0.83_Br_017_)_3_	Heat (30 °C and 70 °C)	ToF-SIMS	[34]
MAPbI_3_	Heat (Oven and plate)	ToF-SIMS	[86]
MAPbBr_3_	Heat	ToF-SIMS	[87]
MAPbBr_3_	Bias (−2~2 V)	ToF-SIMS	[88]
MAPbBr_3_	Bias (0~2 V)	ToF-SIMS	[89]
MAPbI_3_	Bias (~0.5 V)	ToF-SIMS	[90]
MAPbI_3_	Bias	ToF-SIMS	[91]
MAPbI_3_	Water (0 to 10^13^ Langmuir)	XPS, UPS	[97]
MAPbI_3_	Light (408 nm, blue laser, 6.8 mW·cm^−2^)	XPS	[98]
MAPbI_3−x_Cl_x_	Water (10^−6^ mbar,4 mbar), Oxygen (50 mbar), air	UPS	[99]
MAPbI_3_	Water (3 mbar (10% RH) and 9 mbar (30% RH))	NAPXPS	[100]
FAPbI_3_, MAPbI_3_	Water (23 mbar (80% RH))	NAPXPS	[101]
MAPb(I_0.83_Cl_0.17_)_3_	Light (white)	XPS	[102]
(MAFA)PbIBr	Light (515 nm laser, 280 to 2800 mW·cm^−2^)	XPS	[104]

**Table 3:** Summary of in situ/operando techniques applied in morphology structure studies.

Perovskite	Exposure Condition	Techniques	Ref.
MAPbI_3_	Heat (25~170 °C)	TEM, SEM	[110]
MAPbI_3_	Heat (85 °C)	TEM	[35]
MAPbI_3_	Heat (50~250 °C)	TEM	[111]
MAPbI_3_	Heat (50~60 °C)	TEM	[112]
(CsFAMA)_1_Pb(IBr)_3_	Heat (85~170 °C)	TEM	[105]
MAPbI_3_	Light (white LED)	TEM	[118]
(MAFA)_1_Pb(IBr)_3_	Bias (1 V)	TEM	[107]
MAPbI_3_	Bias (−6~6 V)	TEM	[116]
MAPbI_3_	Bias (−1~1 V)	TEM	[117]
MAPbI_3_	Humidity (80%)	AFM	[49]

**Table 4:** Summary of in situ/operando techniques applied in optical properties.

Perovskite Films	Exposure Condition	Techniques	Refs
MAPbI_3_, MAPbBr_3_	Water (80% RH)	PL	[123]
MAPbI_3_	Water (55% RH and 80% RH)	IBIC	[125]
(PEA)_2_PbI_4_	Light (488 nm, 120 mW cm^−2^)	CLSM	[36]
CsPbIBr_2_	Light (470 nm laser, 100 mW cm^−2^)	PL	[126]

## Data Availability

Not applicable.

## References

[1] Bai S., Da P., Li C., Wang Z., Yuan Z., Fu F., Kawecki M., Liu X., Sakai N., Wang J.T. (2019). Planar perovskite solar cells with long-term stability using ionic liquid additives. Nature.

[2] Cao Y., Wang N., Tian H., Guo J., Wei Y., Chen H., Miao Y., Zou W., Pan K., He Y. (2018). Perovskite light-emitting diodes based on spontaneously formed submicrometre-scale structures. Nature.

[3] Lin K., Xing J., Quan L.N., de Arquer F.P.G., Gong X., Lu J., Xie L., Zhao W., Zhang D., Yan C. (2018). Perovskite light-emitting diodes with external quantum efficiency exceeding 20 per cent. Nature.

[4] Shen Y., Shen K.C., Li Y.Q., Guo M., Wang J., Ye Y., Xie F.M., Ren H., Gao X., Song F. (2020). Interfacial Potassium-Guided Grain Growth for Efficient Deep-Blue Perovskite Light-Emitting Diodes. Adv. Funct. Mater..

[5] Li J., Wang J., Ma J., Shen H., Li L., Duan X., Li D. (2019). Self-trapped state enabled filterless narrowband photodetections in 2D layered perovskite single crystals. Nat. Commun..

[6] Dong H., Zhang C., Liu X., Yao J., Zhao Y.S. (2020). Materials chemistry and engineering in metal halide perovskite lasers. Chem. Soc. Rev..

[7] Liu Y., Yang Z., Cui D., Ren X., Sun J., Liu X., Zhang J., Wei Q., Fan H., Yu F. (2015). Two-Inch-Sized Perovskite CH_3_ NH_3_ PbX_3_ (X = Cl, Br, I) Crystals: Growth and Characterization. Adv. Mater..

[8] Huang J., Yuan Y., Shao Y., Yan Y. (2017). Understanding the physical properties of hybrid perovskites for photovoltaic applications. Nat. Rev. Mater..

[9] Dong Q., Fang Y., Shao Y., Mulligan P., Qiu J., Cao L., Huang J. (2015). Solar cells. Electron-hole diffusion lengths > 175 μm in solution-grown CH_3_NH_3_PbI_3_ single crystals. Science.

[10] Stranks S.D., Eperon G.E., Grancini G., Menelaou C., Alcocer M.J.P., Leijtens T., Herz L.M., Petrozza A., Snaith H.J. (2013). Electron-hole diffusion lengths exceeding 1 micrometer in an organometal trihalide perovskite absorber. Science.

[11] Poindexter J.R., Hoye R.L.Z., Nienhaus L., Kurchin R.C., Morishige A.E., Looney E.E., Osherov A., Correa-Baena J.P., Lai B., Bulovic V. (2017). High Tolerance to Iron Contamination in Lead Halide Perovskite Solar Cells. ACS Nano.

[12] Steirer K.X., Schulz P., Teeter G., Stevanovic V., Yang M., Zhu K., Berry J.J. (2016). Defect Tolerance in Methylammonium Lead Triiodide Perovskite. ACS Energy Lett..

[13] Kojima A., Teshima K., Shirai Y., Miyasaka T.J. (2009). Organometal Halide Perovskites as Visible-Light Sensitizers for Photovoltaic Cells. Am. Chem. Soc..

[14] Jeong J., Kim M., Seo J., Lu H., Ahlawat P., Mishra A., Yang Y., Hope M.A., Eickemeyer F.T., Kim M. (2021). Pseudo-halide anion engineering for alpha-FAPbI_3_ perovskite solar cells. Nature.

[15] Park J., Kim J., Yun H.S., Paik M.J., Noh E., Mun H.J., Kim M.G., Shin T.J., Seok S.I. (2023). Controlled growth of perovskite layers with volatile alkylammonium chlorides. Nature.

[16] Klampaftis E., Richards B.S. (2011). Improvement in multi-crystalline silicon solar cell efficiency via addition of luminescent material to EVA encapsulation layer. Prog. Photovolt..

[17] Singh A.N., Kajal S., Kim J., Jana A., Kim J.Y., Kim K.S. (2020). Interface Engineering Driven Stabilization of Halide Perovskites against Moisture, Heat, and Light for Optoelectronic Applications. Adv. Energy Mater..

[18] Béchu S., Ralaiarisoa M., Etcheberry A., Schulz P. (2020). Photoemission Spectroscopy Characterization of Halide Perovskites. Adv. Energy Mater..

[19] Kosasih F.U., Ducati C. (2018). Characterising degradation of perovskite solar cells through in-situ and operando electron microscopy. Nano Energy.

[20] Wei J., Wang Q., Huo J., Gao F., Gan Z., Zhao Q., Li H. (2020). Mechanisms and Suppression of Photoinduced Degradation in Perovskite Solar Cells. Adv. Energy Mater..

[21] Hidalgo J., Castro-Méndez A.F., Correa-Baena J.P. (2019). Imaging and Mapping Characterization Tools for Perovskite Solar Cells. Adv. Energy Mater..

[22] Boyd C.C., Cheacharoen R., Leijtens T., McGehee M.D. (2019). Understanding Degradation Mechanisms and Improving Stability of Perovskite Photovoltaics. Chem. Rev..

[23] Kundu S., Kelly T.L. (2020). In situ studies of the degradation mechanisms of perovskite solar cells. EcoMat.

[24] Zhou Y., Sternlicht H., Padture N.P. (2019). Transmission Electron Microscopy of Halide Perovskite Materials and Devices. Joule.

[25] Szostak R., Goncalves A.d.S., Freitas J.N.d., Marchezi P.E., Araujo F.L.d., Tolentino H.C.N., Toney M.F., Marques F.d.C., Nogueira A.F. (2023). In Situ and Operando Characterizations of Metal Halide Perovskite and Solar Cells: Insights from Lab-Sized Devices to Upscaling Processes. Chem. Rev..

[26] Meng X., Tian X., Zhang S., Zhou J., Zhang Y., Liu Z., Chen W. (2022). In Situ Characterization for Understanding the Degradation in Perovskite Solar Cells. Sol. RRL.

[27] Dunfield S.P., Bliss L., Zhang F., Luther J.M., Zhu K., Hest M.F.A.M., Reese M.O., Berry J.J. (2020). From Defects to Degradation: A Mechanistic Understanding of Degradation in Perovskite Solar Cell Devices and Modules. Adv. Energy Mater..

[28] Zhang S., Liu Z., Zhang W., Jiang Z., Chen W., Chen R., Huang Y., Yang Z., Zhang Y., Han L. (2020). Barrier Designs in Perovskite Solar Cells for Long-Term Stability. Adv. Energy Mater..

[29] Nguyen L., Tao F.F., Tang Y., Dou J., Bao X.-J. (2019). Understanding Catalyst Surfaces during Catalysis through Near Ambient Pressure X-ray Photoelectron Spectroscopy. Chem. Rev..

[30] Chakrabarti A., Ford M.E., Gregory D., Hu R., Keturakis C.J., Lwin S., Tang Y., Yang Z., Zhu M., Bañares M.A. (2017). A decade+ of operando spectroscopy studies. Catal. Today.

[31] Bañares M.A., Guerrero-Pérez M.O., Fierro J.L.G., Cortez G.G. (2002). Raman spectroscopy during catalytic operations with on-line activity measurement (operando spectroscopy): A method for understanding the active centres of cations supported on porous materials. J. Mater. Chem..

[32] Zhao L., Kerner R.A., Xiao Z., Lin Y.L., Lee K.M., Schwartz J., Rand B.P. (2016). Redox Chemistry Dominates the Degradation and Decomposition of Metal Halide Perovskite Optoelectronic Devices. ACS Energy Lett..

[33] Staebler D.L., Crandall R.S., Williams R. (1981). Stability of n-i-p amorphous silicon solar cells *Appl*. Phys. Lett..

[34] Domanski K., Correa-Baena J.P., Mine N., Nazeeruddin M.K., Abate A., Saliba M., Tress W., Hagfeldt A., Gratzel M. (2016). Not All That Glitters Is Gold: Metal-Migration-Induced Degradation in Perovskite Solar Cells. ACS Nano.

[35] Fan Z., Xiao H., Wang Y., Zhao Z., Lin Z., Cheng H.-C., Lee S.-J., Wang G., Feng Z., Goddard W.A. (2017). Layer-by-Layer Degradation of Methylammonium Lead Tri-iodide Perovskite Microplates. Joule.

[36] Fang H.-H., Yang J., Tao S., Adjokatse S., Kamminga M.E., Ye J., Blake G.R., Even J., Loi M.A. (2018). Unravelling Light-Induced Degradation of Layered Perovskite Crystals and Design of Efficient Encapsulation for Improved Photostability. Adv. Funct. Mater..

[37] Yin J., Teobaldi G., Liu L.M. (2022). The Role of Thermal Fluctuations and Vibrational Entropy: A Theoretical Insight into the delta-to-alpha Transition of FAPbI(3). J. Phys. Chem. Lett..

[38] Niu T., Chao L., Dong X., Fu L., Chen Y. (2022). Phase-Pure α-FAPbI3 for Perovskite Solar Cells. J. Phys. Chem. Lett..

[39] Masi S., Gualdrón-Reyes A.F., Mora-Seró I. (2020). Stabilization of Black Perovskite Phase in FAPbI_3_ and CsPbI_3_. ACS Energy Lett..

[40] Su Z., Wang C., Zheng G., Gao X. (2021). Impacts of MAPbBr_3_ Additive on Crystallization Kinetics of _FAPbI3_ Perovskite for High Performance Solar Cells. Coatings.

[41] Li G., Su Z., Canil L., Hughes D., Aldamasy M.H., Dagar J., Trofimov S., Wang L., Zuo W., Jerónimo-Rendon J.J. (2023). Highly efficient p-i-n perovskite solar cells that endure temperature variations. Science.

[42] Niu G., Li W., Meng F., Wang L., Dong H., Qiu Y. (2014). Study on the stability of CH_3_NH_3_PbI_3_ films and the effect of post-modification by aluminum oxide in all-solid-state hybrid solar cells. J. Mater. Chem. A.

[43] Christians J.A., Miranda Herrera P.A., Kamat P.V. (2015). Transformation of the excited state and photovoltaic efficiency of CH_3_NH_3_PbI_3_ perovskite upon controlled exposure to humidified air. J. Am. Chem. Soc..

[44] Zhang M., Zhang F., Wang Y., Zhu L., Hu Y., Lou Z., Hou Y., Teng F. (2018). High-Performance Photodiode-Type Photodetectors Based on Polycrystalline Formamidinium Lead Iodide Perovskite Thin Films. Sci. Rep..

[45] Yang J., Siempelkamp B.D., Liu D., Kelly T.L. (2015). Investigation of CH_3_NH_3_PbI_3_ degradation rates and mechanisms in controlled humidity environments using in situ techniques. ACS Nano.

[46] Fransishyn K.M., Kundu S., Kelly T.L. (2018). Elucidating the Failure Mechanisms of Perovskite Solar Cells in Humid Environments Using In Situ Grazing-Incidence Wide-Angle X-ray Scattering. ACS Energy Lett..

[47] Chen B.-A., Lin J.-T., Suen N.-T., Tsao C.-W., Chu T.-C., Hsu Y.-Y., Chan T.-S., Chan Y.-T., Yang J.-S., Chiu C.-W. (2017). In Situ Identification of Photo- and Moisture-Dependent Phase Evolution of Perovskite Solar Cells. ACS Energy Lett..

[48] Leguy A.M.A., Hu Y., Campoy-Quiles M., Alonso M.I., Weber O.J., Azarhoosh P., van Schilfgaarde M., Weller M.T., Bein T., Nelson J. (2015). Reversible Hydration of CH_3_NH_3_PbI_3_ in Films, Single Crystals, and Solar Cells. Chem. Mater..

[49] Li D., Bretschneider S.A., Bergmann V.W., Hermes I.M., Mars J., Klasen A., Lu H., Tremel W., Mezger M., Butt H.-J. (2016). Humidity-Induced Grain Boundaries in MAPbI_3_ Perovskite Films. J. Phys. Chem. C.

[50] Schlipf J., Biessmann L., Oesinghaus L., Berger E., Metwalli E., Lercher J.A., Porcar L., Muller-Buschbaum P. (2018). In Situ Monitoring the Uptake of Moisture into Hybrid Perovskite Thin Films. J. Phys. Chem. Lett..

[51] Long M., Zhang T., Liu M., Chen Z., Wang C., Xie W., Xie F., Chen J., Li G., Xu J. (2018). Abnormal Synergetic Effect of Organic and Halide Ions on the Stability and Optoelectronic Properties of a Mixed Perovskite via In Situ Characterizations. Adv. Mater..

[52] Tan W., Bowring A.R., Meng A.C., McGehee M.D., McIntyre P.C. (2018). Thermal Stability of Mixed Cation Metal Halide Perovskites in Air. ACS Appl. Mater. Interfaces.

[53] Nenon D.P., Christians J.A., Wheeler L.M., Blackburn J.L., Sanehira E.M., Dou B., Olsen M.L., Zhu K., Berry J.J., Luther J.M. (2016). Structural and chemical evolution of methylammonium lead halide perovskites during thermal processing from solution. Energy Environ. Sci..

[54] Sutter-Fella C.M., Ngo Q.P., Cefarin N., Gardner K.L., Tamura N., Stan C.V., Drisdell W.S., Javey A., Toma F.M., Sharp I.D. (2018). Cation-Dependent Light-Induced Halide Demixing in Hybrid Organic-Inorganic Perovskites. Nano Lett..

[55] Li M., Wang Z.-K., Yang Y.-G., Hu Y., Feng S.-L., Wang J.-M., Gao X.-Y., Liao L.-S. (2016). Copper Salts Doped Spiro-OMeTAD for High-Performance Perovskite Solar Cells. Adv. Energy Mater..

[56] Miyadera T., Shibata Y., Koganezawa T., Murakami T.N., Sugita T., Tanigaki N., Chikamatsu M. (2015). Crystallization Dynamics of Organolead Halide Perovskite by Real-Time X-ray Diffraction. Nano Lett..

[57] Wang Y., Dar M.I., Ono L.K., Zhang T., Kan M., Li Y., Zhang L., Wang X., Yang Y., Gao X. (2019). Thermodynamically stabilized β-CsPbI_3_-based perovskite solar cells with efficiencies. Science.

[58] Qin M., Tse K., Lau T.K., Li Y., Su C.J., Yang G., Chen J., Zhu J., Jeng U.S., Li G. (2019). Manipulating the Mixed-Perovskite Crystallization Pathway Unveiled by In Situ GIWAXS. Adv. Mater..

[59] Lu L., Shen K.C., Wang J., Su Z., Li Y., Chen L., Luo Y., Song F., Gao X., Tang J.X. (2020). Interaction of the Cation and Vacancy in Hybrid Perovskites Induced by Light Illumination. ACS Appl. Mater. Interfaces.

[60] Song J., Zhou G., Chen W., Zhang Q., Ali J., Hu Q., Wang J., Wang C., Feng W., Djurisic A.B. (2020). Unraveling the Crystallization Kinetics of 2D Perovskites with Sandwich-Type Structure for High-Performance Photovoltaics. Adv. Mater..

[61] Qin M., Chan P.F., Lu X. (2021). A Systematic Review of Metal Halide Perovskite Crystallization and Film Formation Mechanism Unveiled by In Situ GIWAXS. Adv. Mater..

[62] Schlipf J., Hu Y., Pratap S., Bießmann L., Hohn N., Porcar L., Bein T., Docampo P., Müller-Buschbaum P. (2019). Shedding Light on the Moisture Stability of 3D/2D Hybrid Perovskite Heterojunction Thin Films. ACS Appl. Energy Mater..

[63] Hada M., Hasegawa Y., Nagaoka R., Miyake T., Abdullaev U., Ota H., Nishikawa T., Yamashita Y., Hayashi Y. (2018). In-situ X-ray diffraction reveals the degradation of crystalline CH_3_NH_3_PbI_3_ by water-molecule collisions at room temperature. Jpn. J. Appl. Phys..

[64] Pistor P., Burwig T., Brzuska C., Weber B., Fränzel W. (2018). Thermal stability and miscibility of co-evaporated methyl ammonium lead halide (MAPbX3, X = I, Br, Cl) thin films analysed by in situ X-ray diffraction. J. Mater. Chem. A.

[65] Tang X., Brandl M., May B., Levchuk I., Hou Y., Richter M., Chen H., Chen S., Kahmann S., Osvet A. (2016). Photoinduced degradation of methylammonium lead triiodide perovskite semiconductors. J. Mater. Chem. A.

[66] Ruf F., Rietz P., Aygüler M.F., Kelz I., Docampo P., Kalt H., Hetterich M. (2018). The Bandgap as a Moving Target: Reversible Bandgap Instabilities in Multiple-Cation Mixed-Halide Perovskite Solar Cells. ACS Energy Lett..

[67] Barker A.J., Sadhanala A., Deschler F., Gandini M., Senanayak S.P., Pearce P.M., Mosconi E., Pearson A.J., Wu Y., Srimath Kandada A.R. (2017). Defect-Assisted Photoinduced Halide Segregation in Mixed-Halide Perovskite Thin Films. ACS Energy Lett..

[68] Kim N.K., Min Y.H., Noh S., Cho E., Jeong G., Joo M., Ahn S.W., Lee J.S., Kim S., Ihm K. (2017). Investigation of Thermally Induced Degradation in CH_3_NH_3_PbI_3_ Perovskite Solar Cells using In-situ Synchrotron Radiation Analysis. Sci. Rep..

[69] Juarez-Perez E.J., Hawash Z., Raga S.R., Ono L.K., Qi Y. (2016). Thermal degradation of CH_3_NH_3_PbI_3_ perovskite into NH_3_ and CH_3_I gases observed by coupled thermogravimetry–mass spectrometry analysis. Energy Environ. Sci..

[70] Juarez-Perez E.J., Ono L.K., Qi Y. (2019). Thermal degradation of formamidinium based lead halide perovskites into sym-triazine and hydrogen cyanide observed by coupled thermogravimetry-mass spectrometry analysis. J. Mater. Chem. A.

[71] Williams A.E., Holliman P.J., Carnie M.J., Davies M.L., Worsley D.A., Watson T.M. (2014). Perovskite processing for photovoltaics: A spectro-thermal evaluation. J. Mater. Chem. A.

[72] Ciccioli A., Latini A. (2018). Thermodynamics and the Intrinsic Stability of Lead Halide Perovskites CH_3_NH_3_PbX_3_. J. Phys. Chem. Lett..

[73] Xu D., Hua X., Liu S.C., Qiao H.W., Yang H.G., Long Y.T., Tian H. (2018). In situ and real-time ToF-SIMS analysis of light-induced chemical changes in perovskite CH_3_NH_3_PbI_3_. Chem. Commun..

[74] Nickel N.H., Lang F., Brus V.V., Shargaieva O., Rappich J. (2017). Unraveling the Light-Induced Degradation Mechanisms of CH_3_NH_3_PbI_3_ Perovskite Films. Adv. Electron. Mater..

[75] Juarez-Perez E.J., Ono L.K., Maeda M., Jiang Y., Hawash Z., Qi Y. (2018). Photodecomposition and thermal decomposition in methylammonium halide lead perovskites and inferred design principles to increase photovoltaic device stability. J. Mater. Chem. A.

[76] Song Z., Wang C., Phillips A.B., Grice C.R., Zhao D., Yu Y., Chen C., Li C., Yin X., Ellingson R.J. (2018). Probing the origins of photodegradation in organic–inorganic metal halide perovskites with time-resolved mass spectrometry. Sustain. Energy Fuels.

[77] Zhang T., Cheung S.H., Meng X., Zhu L., Bai Y., Ho C.H.Y., Xiao S., Xue Q., So S.K., Yang S. (2017). Pinning Down the Anomalous Light Soaking Effect toward High-Performance and Fast-Response Perovskite Solar Cells: The Ion-Migration-Induced Charge Accumulation. J. Phys. Chem. Lett..

[78] Duong T., Wu Y., Shen H., Peng J., Zhao S., Wu N., Lockrey M., White T., Weber K., Catchpole K. (2018). Light and elevated temperature induced degradation (LeTID) in perovskite solar cells and development of stable semi-transparent cells. Sol. Energy Mat. Sol. Cells.

[79] Lin W.-C., Chang H.-Y., Abbasi K., Shyue J.-J., Burda C. (2017). 3D In Situ ToF-SIMS Imaging of Perovskite Films under Controlled Humidity Environmental Conditions. Adv. Mater. Interfaces.

[80] Xu D., Liu D., Xie T., Cao Y., Wang J.G., Ning Z.J., Long Y.T., Tian H. (2016). Plasmon resonance scattering at perovskite CH_3_NH_3_PbI_3_ coated single gold nanoparticles: Evidence for electron transfer. Chem. Commun..

[81] Aristidou N., Eames C., Islam M.S., Haque S.A. (2017). Insights into the increased degradation rate of CH_3_NH_3_PbI_3_ solar cells in combined water and O2 environments. J. Mater. Chem. A.

[82] Aristidou N., Eames C., Sanchez-Molina I., Bu X., Kosco J., Islam M.S., Haque S.A. (2017). Fast oxygen diffusion and iodide defects mediate oxygen-induced degradation of perovskite solar cells. Nat. Commun..

[83] Senocrate A., Acartürk T., Kim G.Y., Merkle R., Starke U., Grätzel M., Maier J. (2018). Interaction of oxygen with halide perovskites. J. Mater. Chem. A.

[84] Wygant B.R., Ye A.Z., Dolocan A., Vu Q., Abbot D.M., Mullins C.B. (2019). Probing the Degradation Chemistry and Enhanced Stability of 2D Organolead Halide Perovskites. J. Am. Chem. Soc..

[85] Li J., Dong Q., Li N., Wang L. (2017). Direct Evidence of Ion Diffusion for the Silver-Electrode-Induced Thermal Degradation of Inverted Perovskite Solar Cells. Adv. Energy Mater..

[86] Wang X., Liu H., Zhou F., Dahan J., Wang X., Li Z., Shen W. (2018). Temperature Gradient-Induced Instability of Perovskite via Ion Transport. ACS Appl. Mater. Interfaces.

[87] Palma A.L., Cina L., Busby Y., Marsella A., Agresti A., Pescetelli S., Pireaux J.J., Di Carlo A. (2016). Mesoscopic Perovskite Light-Emitting Diodes. ACS Appl. Mater. Interfaces.

[88] Domanski K., Roose B., Matsui T., Saliba M., Turren-Cruz S.-H., Correa-Baena J.-P., Carmona C.R., Richardson G., Foster J.M., De Angelis F. (2017). Migration of cations induces reversible performance losses over day/night cycling in perovskite solar cells. Energy Environ. Sci..

[89] Lee H., Ko D., Lee C. (2019). Direct Evidence of Ion-Migration-Induced Degradation of Ultrabright Perovskite Light-Emitting Diodes. ACS Appl. Mater. Interfaces.

[90] Zhao Y., Zhou W., Tan H., Fu R., Li Q., Lin F., Yu D., Walters G., Sargent E.H., Zhao Q. (2017). Mobile-Ion-Induced Degradation of Organic Hole-Selective Layers in Perovskite Solar Cells. J. Phys. Chem. C.

[91] Lee H., Lee C. (2018). Analysis of Ion-Diffusion-Induced Interface Degradation in Inverted Perovskite Solar Cells via Restoration of the Ag Electrode. Adv. Energy Mater..

[92] Drozdov M.N., Yunin P.A., Travkin V.V., Koptyaev A.I., Pakhomov G.L. (2019). Direct Imaging of Current-Induced Transformation of a Perovskite/Electrode Interface. Adv. Mater. Interfaces.

[93] Wang J., Senanayak S.P., Liu J., Hu Y., Shi Y., Li Z., Zhang C., Yang B., Jiang L., Di D. (2019). Investigation of Electrode Electrochemical Reactions in CH3 NH3 PbBr3 Perovskite Single-Crystal Field-Effect Transistors. Adv. Mater..

[94] Bridges C.A., Sun X.-G., Zhao J., Paranthaman M.P., Dai S. (2012). In Situ Observation of Solid Electrolyte Interphase Formation in Ordered Mesoporous Hard Carbon by Small-Angle Neutron Scattering. J. Phys. Chem. C.

[95] Huang W., Manser J.S., Kamat P.V., Ptasinska S. (2015). Evolution of Chemical Composition, Morphology, and Photovoltaic Efficiency of CH_3_NH_3_PbI_3_ Perovskite under Ambient Conditions. Chem. Mater..

[96] Philippe B., Park B.-W., Lindblad R., Oscarsson J., Ahmadi S., Johansson E.M.J., Rensmo H. (2015). Chemical and Electronic Structure Characterization of Lead Halide Perovskites and Stability Behavior under Different Exposures—A Photoelectron Spectroscopy Investigation. Chem. Mater..

[97] Li Y., Xu X., Wang C., Wang C., Xie F., Yang J., Gao Y. (2015). Degradation by Exposure of Coevaporated CH_3_NH_3_PbI_3_ Thin Films. J. Phys. Chem. C.

[98] Li Y., Xu X., Wang C., Ecker B., Yang J., Huang J., Gao Y. (2017). Light-Induced Degradation of CH_3_NH_3_PbI_3_ Hybrid Perovskite Thin Film. J. Phys. Chem. C.

[99] Ralaiarisoa M., Salzmann I., Zu F.S., Koch N. (2018). Effect of Water, Oxygen, and Air Exposure on CH_3_NH_3_PbI_3–x_Cl_x_ Perovskite Surface Electronic Properties. Adv. Electron. Mater..

[100] Chun-Ren Ke J., Walton A.S., Lewis D.J., Tedstone A., O’Brien P., Thomas A.G., Flavell W.R. (2017). In situ investigation of degradation at organometal halide perovskite surfaces by X-ray photoelectron spectroscopy at realistic water vapour pressure. Chem. Commun..

[101] Chen S., Solanki A., Pan J., Sum T.C. (2019). Compositional and Morphological Changes in Water-Induced Early-Stage Degradation in Lead Halide Perovskites. Coatings.

[102] Yang J., Hong Q., Yuan Z., Xu R., Guo X., Xiong S., Liu X., Braun S., Li Y., Tang J. (2018). Unraveling Photostability of Mixed Cation Perovskite Films in Extreme Environment. Adv. Opt. Mater..

[103] Xu R.P., Li Y.Q., Jin T.Y., Liu Y.Q., Bao Q.Y., O’Carroll C., Tang J.X. (2018). In Situ Observation of Light Illumination-Induced Degradation in Organometal Mixed-Halide Perovskite Films. ACS Appl. Mater. Interfaces.

[104] Cappel U.B., Svanstrom S., Lanzilotto V., Johansson F.O.L., Aitola K., Philippe B., Giangrisostomi E., Ovsyannikov R., Leitner T., Fohlisch A. (2017). Partially Reversible Photoinduced Chemical Changes in a Mixed-Ion Perovskite Material for Solar Cells. ACS Appl. Mater. Interfaces.

[105] Seo Y.-H., Kim J.H., Kim D.-H., Chung H.-S., Na S.-I. (2020). In situ TEM observation of the heat–induced degradation of single—and triple–cation planar perovskite solar cells. Nano Energy.

[106] Aguiar J.A., Wozny S., Alkurd N.R., Yang M., Kovarik L., Holesinger T.G., Al-Jassim M., Zhu K., Zhou W., Berry J.J. (2016). Effect of Water Vapor, Temperature, and Rapid Annealing on Formamidinium Lead Triiodide Perovskite Crystallization. ACS Energy Lett..

[107] Kim M.-c., Ahn N., Cheng D., Xu M., Ham S.-Y., Pan X., Kim S.J., Luo Y., Fenning D.P., Tan D.H.S. (2021). Imaging Real-Time Amorphization of Hybrid Perovskite Solar Cells under Electrical Biasing. ACS Energy Lett..

[108] Aguiar J.A., Wozny S., Holesinger T.G., Aoki T., Patel M.K., Yang M., Berry J.J., Al-Jassim M., Zhou W., Zhu K. (2016). In situ investigation of the formation and metastability of formamidinium lead tri-iodide perovskite solar cells. Energy Environ. Sci..

[109] Qin F., Wang Z., Wang Z.L. (2016). Anomalous Growth and Coalescence Dynamics of Hybrid Perovskite Nanoparticles Observed by Liquid-Cell Transmission Electron Microscopy. ACS Nano.

[110] Kim T.W., Shibayama N., Cojocaru L., Uchida S., Kondo T., Segawa H. (2018). Real-Time In Situ Observation of Microstructural Change in Organometal Halide Perovskite Induced by Thermal Degradation. Adv. Funct. Mater..

[111] Divitini G., Cacovich S., Matteocci F., Cinà L., Di Carlo A., Ducati C. (2016). In situ observation of heat-induced degradation of perovskite solar cells. Nat. Energy.

[112] Yang B., Dyck O., Ming W., Du M.H., Das S., Rouleau C.M., Duscher G., Geohegan D.B., Xiao K. (2016). Observation of Nanoscale Morphological and Structural Degradation in Perovskite Solar Cells by in Situ TEM. ACS Appl. Mater. Interfaces.

[113] Azpiroz J.M., Mosconi E., Bisquert J., De Angelis F. (2015). Defect migration in methylammonium lead iodide and its role in perovskite solar cell operation. Energy Environ. Sci..

[114] Eames C., Frost J.M., Barnes P.R., O’Regan B.C., Walsh A., Islam M.S. (2015). Ionic transport in hybrid lead iodide perovskite solar cells. Nat. Commun..

[115] Haruyama J., Sodeyama K., Han L., Tateyama Y. (2015). First-Principles Study of Ion Diffusion in Perovskite Solar Cell Sensitizers. J. Am. Chem. Soc..

[116] Jeangros Q., Duchamp M., Werner J., Kruth M., Dunin-Borkowski R.E., Niesen B., Ballif C., Hessler-Wyser A. (2016). In Situ TEM Analysis of Organic-Inorganic Metal-Halide Perovskite Solar Cells under Electrical Bias. Nano Lett..

[117] Jung H.J., Kim D., Kim S., Park J., Dravid V.P., Shin B. (2018). Stability of Halide Perovskite Solar Cell Devices: In Situ Observation of Oxygen Diffusion under Biasing. Adv. Mater..

[118] Lu Y., Hu J., Ge Y., Tian B., Zhang Z., Sui M. (2021). Decisive influence of amorphous PbI2−x on the photodegradation of halide perovskites. J. Mater. Chem. A.

[119] Ran J., Dyck O., Wang X., Yang B., Geohegan D.B., Xiao K. (2020). Electron-Beam-Related Studies of Halide Perovskites: Challenges and Opportunities. Adv. Energy Mater..

[120] Kutes Y., Zhou Y., Bosse J.L., Steffes J., Padture N.P., Huey B.D. (2016). Mapping the Photoresponse of CH_3_NH_3_PbI_3_ Hybrid Perovskite Thin Films at the Nanoscale. Nano Lett..

[121] Wang Q., Chen B., Liu Y., Deng Y., Bai Y., Dong Q., Huang J. (2017). Scaling behavior of moisture-induced grain degradation in polycrystalline hybrid perovskite thin films. Energy Environ. Sci..

[122] Ji F., Pang S., Zhang L., Zong Y., Cui G., Padture N.P., Zhou Y. (2017). Simultaneous Evolution of Uniaxially Oriented Grains and Ultralow-Density Grain-Boundary Network in CH_3_NH_3_PbI_3_ Perovskite Thin Films Mediated by Precursor Phase Metastability. ACS Energy Lett..

[123] Song Z., Shrestha N., Watthage S.C., Liyanage G.K., Almutawah Z.S., Ahangharnejhad R.H., Phillips A.B., Ellingson R.J., Heben M.J. (2018). Impact of Moisture on Photoexcited Charge Carrier Dynamics in Methylammonium Lead Halide Perovskites. J. Phys. Chem. Lett..

[124] Chen W., Gan Z., Green M.A., Jia B., Wen X. (2020). Revealing Dynamic Effects of Mobile Ions in Halide Perovskite Solar Cells Using Time-Resolved Microspectroscopy. Small Methods.

[125] Song Z., Abate A., Watthage S.C., Liyanage G.K., Phillips A.B., Steiner U., Graetzel M., Heben M.J. (2016). Perovskite Solar Cell Stability in Humid Air: Partially Reversible Phase Transitions in the PbI_2_-CH_3_NH_3_H_2_O System. Adv. Energy Mater..

[126] Yin X., Guo Y., Liu J., Que W., Ma F., Xu K. (2020). Photoinduced Phase Segregation Leading to Evident Open-Circuit Voltage Loss in Efficient Inorganic CsPbIBr2 Solar Cells. J. Phys. Chem. Lett..

[127] Liu S., Guan Y., Sheng Y., Hu Y., Rong Y., Mei A., Han H. (2019). A Review on Additives for Halide Perovskite Solar Cells. Adv. Energy Mater..

[128] Gao F., Zhao Y., Zhang X., You J. (2019). Recent Progresses on Defect Passivation toward Efficient Perovskite Solar Cells. Adv. Energy Mater..

[129] Akin S., Arora N., Zakeeruddin S.M., Grätzel M., Friend R.H., Dar M.I. (2019). New Strategies for Defect Passivation in High-Efficiency Perovskite Solar Cells. Adv. Energy Mater..

[130] Mahmud M.A., Duong T., Peng J., Wu Y., Shen H., Walter D., Nguyen H.T., Mozaffari N., Tabi G.D., Catchpole K.R. (2021). Origin of Efficiency and Stability Enhancement in High-Performing Mixed Dimensional 2D-3D Perovskite Solar Cells: A Review. Adv. Funct. Mater..

[131] Wang S., Zhang Z., Tang Z., Su C., Huang W., Li Y., Xing G. (2021). Polymer strategies for high-efficiency and stable perovskite solar cells. Nano Energy.

[132] Zhang F., Zhu K. (2019). Additive Engineering for Efficient and Stable Perovskite Solar Cells. Adv. Energy Mater..

[133] Grancini G., Roldan-Carmona C., Zimmermann I., Mosconi E., Lee X., Martineau D., Narbey S., Oswald F., De Angelis F., Graetzel M. (2017). One-Year stable perovskite solar cells by 2D/3D interface engineering. Nat. Commun..

